# National assessment of Canadian pandemic preparedness: Employing InFluNet to identify high-risk areas for inter-wave vaccine distribution

**DOI:** 10.1016/j.idm.2017.06.005

**Published:** 2017-07-05

**Authors:** Patrick Saunders-Hastings, Bryson Quinn Hayes, Robert Smith？, Daniel Krewski

**Affiliations:** a University of Ottawa, McLaughlin Centre for Population Health Risk Assessment, 850 Peter Morand Crescent, Ottawa, Ontario, K1G 5Z3, Canada; b University of Ottawa, School of Epidemiology, Public Health, and Preventive Medicine, Faculty of Medicine, 451 Smyth Road, Ottawa, ON, K1H 8M5, Canada; c University of Ottawa, Department of Mathematics, 585 King Edward Avenue, Ottawa, ON, K1N 6N5, Canada; d Risk Sciences International, 55 Metcalfe Street, Suite 700, Ottawa, ON, K1P 6L5, Canada

**Keywords:** Pandemic influenza, Vaccination, Differential equations, Mathematical modelling, Surge capacity, Canada

## Abstract

**Background:**

Influenza pandemics emerge at irregular and unpredictable intervals to cause substantial health, economic and social burdens. Optimizing health-system response is vital to mitigating the consequences of future pandemics.

**Methods:**

We developed a mathematical model to assess the preparedness of Canadian health systems to accommodate pandemic-related increases in patient demand. We identify vulnerable areas, assess the potential of inter-wave vaccination to mitigate impacts and evaluate the association between demographic and health-system characteristics in order to identify predictors of pandemic consequences.

**Results:**

Modelled average attack rates were 23.7–37.2% with no intervention and 2.5–6.4% with pre-vaccination. Peak acute-care demand was 7.5–19.5% of capacity with no intervention and 0.6–2.6% with pre-vaccination. The peak ICU demand was 39.3–101.8% with no intervention and 2.9–13.3% with pre-vaccination. Total mortality was 2258–7944 with no intervention and 88–472 with pre-vaccination. Regions of Southern Ontario were identified as most vulnerable to surges in patient demand. The strongest predictors of peak acute-care demand and ICU demand were acute-care bed capacity (R = −0.8697; r^2^ = 0.7564) and ICU bed capacity (R = −0.8151; r^2^ = 0.6644), respectively. Demographic characteristics had mild associations with predicted pandemic consequences.

**Conclusion:**

Inter-wave vaccination provided adequate acute-care resource protection under all scenarios; ICU resource adequacy was protected under mild disease assumptions, but moderate and severe diseases caused demand to exceed expected availability in 21% and 49% of study areas, respectively. Our study informs priority vaccine distribution strategies for pandemic planning, emphasizing the need for targeted early vaccine distribution to high-risk individuals and areas.

## 1 Introduction

In response to widespread global transmission of the A(H1N1) influenza virus, the World Health Organization declared a pandemic on June 11, 2009; this marked the fourth time in one hundred years that a novel influenza virus had emerged to cause significant social, economic and health burdens (Saunders-Hastings & Krewski, 2016). Influenza is an RNA virus that causes annual outbreaks of acute respiratory infections (Fiore et al., 2008). With a high mutation rate preventing substantial accumulation of natural immunity, influenza is the most deadly vaccine-preventable disease in North America (Fiore et al., 2008).

Influenza pandemics result from the emergence of new viral strains to which humans possess no appreciable immunity. This tends to be the result of a process called *antigenic shift*, wherein viral components from different sources interact and combine to form a new viral genotype; if this strain can transmit easily between human hosts and results in illness, a pandemic may emerge. The combined burden of the past four occurrences — the Spanish flu (1918), Asian flu (1957), Hong Kong flu (1968) and Swine flu (2009) — amount to tens of millions of infections, hospitalizations and deaths (Saunders-Hastings & Krewski, 2016). In each case, the pandemic evolved in multiple successive waves, with the second often being more severe than the first (Saunders-Hastings & Krewski, 2016).

Of particular concern in pandemic situations is the expected surge in patient demand, and the resulting strain on hospital-resource capacity. Hospitals tend to rely on just-in-time resource supply, and have limited surge capacity (Saunders-Hastings, Reisman, & Krewski, 2016). Sudden increases in patient demand could quickly overwhelm hospital capacity, leading to dangerous disruptions in service delivery (Oshitani, Kamigaki, & Suzuki, 2008). A key component of pandemic planning must therefore be the identification and support of vulnerable health systems in order to protect hospital-resource adequacy.

Vaccination has been identified as the most cost-effective method of containing pandemic influenza transmission and mitigating its associated burdens (Yang et al., 2009). However, the production, development and distribution of a new pandemic vaccine could take up to six months, therefore making it may unavailable to affect the first wave of a pandemic (Longini, Halloran, Nizam, & Yang, 2004). However, strategic allocation of a limited pandemic vaccine supply during the inter-wave period could help mitigate the threat of a problematic second wave. While an important component of this effort will be the targeting of high-risk individuals, strategic allocation should also involve the targeting of individuals within health systems at greatest risk of being overwhelmed by surges in patient demand.

In this article, we present the findings of modelling simulations for each Canadian Census Metropolitan Area (CMA). Using InFluNet — a mathematical model developed to predict the evolution and impacts of a pandemic influenza outbreak — we project the possible second-wave pandemic burden for each location under various vaccination and disease severity assumptions. Across six health outcomes, we identify areas at greatest risk from an influenza pandemic and identify high-priority areas for inter-wave vaccine allocation. While of particular relevance to Canadian contexts, this research also provides valuable insights for international pandemic preparedness by evaluating on the characteristics of demographic and health-system profiles that underlie regional pandemic influenza vulnerability.

## 2 Methods

The present study relied on InFluNet — a validated differential equation model developed by the authors — to conduct its model simulations. Below, we provide a brief summary of its underlying assumptions and how it was employed to identify vulnerable Canadian hospital systems.

### 2.1 Social contact

InFluNet stratifies the population by age according to the following five groups: infant (0–4), child (5–18), young adult (19–29), adult (30–64) and senior (65 and over). Individuals interact in the household, school or workplace (depending on age) and community, for twelve, eight, and four hours each day, respectively. Individuals will interact preferentially within and across age groups, depending on their age and location. Estimates of location-based contact rates rely on previously published, empirical age-specific data from the United States (Del Valle, Hyman, Hethcote, & Eubank, 2007), combined with time-location divisions as reported by Statistics Canada (StatsCan, 2010). A fixed timestep of four hours was used, as manual verification showed that shorter timesteps did not produce significantly different results. Contact rates by age and location are identical to those presented in Table 1.

**Table 1:** Average number of daily contacts by age group per person per day (Del Valle et al., 2007).

	Infant	Child	Young adult	Adult	Senior	Total
Infant	0.9511	3.5509	1.6740	4.8698	0.6594	11.7052
Child	1.2237	7.3670	1.6153	3.5244	0.6363	14.3668
Young adult	0.6096	1.7070	6.7059	12.1926	1.3209	22.5359
Adult	0.6195	1.3010	4.2591	12.6380	1.4094	20.2271
Senior	0.3498	0.9794	1.9239	5.8766	2.1827	11.3124

These daily totals are further divided into location-specific and age-stratified contact tables, through estimation of how the location-specific frequency and intimacy of interaction will vary between age groups (Table 2, Table 3, Table 4). These estimates are used to generate hourly contact rates, which determine the number of effective contacts (Bansal, Pourbohloul, Hupert, Grenfell, & Meyers, 2010).

**Table 2:** Number of contacts by age group per day (household).

	Infant	Child	Young adult	Adult	Senior	Total
Infant	0.6658	2.4856	1.1718	3.4088	0.5276	8.2596
Child	0.8566	1.8417	1.1307	2.4671	0.5091	6.8053
Young adult	0.4267	1.1949	1.3412	1.8289	0.5283	5.3200
Adult	0.4337	0.9107	0.6389	3.7914	0.7047	6.4793
Senior	0.2798	0.7835	0.7695	2.9383	1.3096	6.0808

**Table 3:** Number of contacts by age group per day (school and workplace).

	Infant	Child	Young adult	Adult	Senior	Total
Infant	0.1427	0.5326	0.2511	0.7305	0.0659	1.7228
Child	0.1836	4.4202	0.2423	0.5287	0.0636	5.4383
Young adult	0.0914	0.2560	3.3530	7.9252	0.3302	11.9558
Adult	0.0929	0.1951	2.7684	5.0552	0.3523	8.4641
Senior	0.0350	0.0979	0.4810	1.4691	0.3274	2.4104

**Table 4:** Number of contacts by age group per day (community).

	Infant	Child	Young adult	Adult	Senior	Total
Infant	0.1427	0.5326	0.2511	0.7305	0.0659	1.7228
Child	0.1836	1.1050	0.2423	0.5287	0.0636	2.1232
Young adult	0.0914	0.2560	2.0118	2.4385	0.4623	5.2601
Adult	0.0929	0.1951	0.8518	3.7914	0.3523	5.2837
Senior	0.0350	0.0979	0.6734	1.4691	0.5457	2.8211

### 2.2 Transmissibility

The model uses a next-generation operator approach to disease transmission, which has been described in detail in previous publications modelling disease in heterogeneous populations (Del Valle et al., 2013, van den Driessche and Watmough, 2002). This method assumes that the disease-transmission rate (β) depends on six parameters: number of effective contacts (γ), susceptibility (α), infectivity (η), duration of contacts (σ), mean number of transmission events per unit time (τ) and the proportion of the population that is either symptomatically (I_C_/N) or asymptomatically (I_A_/N) infected. Equations are presented below, with specific transmissibility function parameters included in Table 5. The transmission rate is

β = β_C_ + β_A_,

where

$${\text{β}}_{\text{C}}=\text{γ}\cdot {\text{α}}_{C}\cdot {\text{η}}_{C}\left(1-e^{-\sigma \cdot t}\right)\cdot \frac{I_{C}}{N}$$

and

$${\text{β}}_{\text{A}}=\text{γ}\cdot {\text{α}}_{A}\cdot {\text{η}}_{A}\cdot \left(1-e^{-\sigma \cdot t}\right)\cdot \frac{I_{A}}{N}.$$

**Table 5:** Transmissibility function parameters.

Symbol	Definition	Sample value	References	Range
γ	Number of effective contacts	As per contact tables	(Del Valle et al., 2013)	0.01–10 (contacts/day)
α	Susceptibility	0.66 for infants 0.47 for children 0.74 for young adults 0.89 for adults; 0.98 for seniors	(Achonu et al., 2011, Reed et al., 2012)	0–1
η	Infectivity	1.0	Assumed	0–1
σ	Duration of contacts	As per contact tables	(Del Valle et al., 2013)	1/2–1/6 (days/contact)
τ	Mean number of transmission events per unit time	0.275; 0.3	(Biggerstaff, Cauchemez, Reed, Gambhir, & Finelli, 2014)	0.17–0.42
$\frac{I_{A}}{N}$	Proportion of population that is infected	Model-generated	NA	0-10%

The contact parameters were drawn from empirical studies. Susceptibility has been reduced to reflect the pre-existing natural immunity that could reasonably be expected in an inter-wave period; estimates are based on empirical data on infection-driven immunity following the first wave of the A(H1N1) pandemic in the United States (Reed et al., 2012) and Canada (Achonu et al., 2011). We assume that unvaccinated infected individuals will be maximally infectious, as we found no reliable data to support recalibration. It should be noted that duration of contact is a measure of the intimacy of each contact, with contact with an infectious individual in the household presenting a higher transmission potential than more transient contacts in school, workplace or community settings. The number of transmission events were adjusted to estimate a pandemic strain of transmissibility equivalent to the moderately transmissible 1957 pandemic (0.275) and highly transmissible 1918 pandemic (0.3) (Biggerstaff et al., 2014).

### 2.3 Model structure

**Fig. 1:**
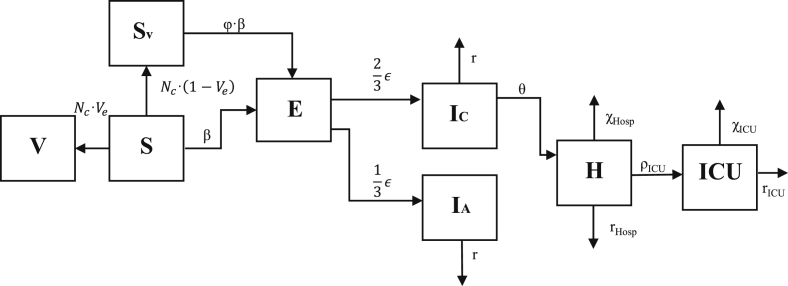
InFluNet transmission model flow diagram.

InFluNet is a deterministic SEIR (susceptible-exposed-infected-recovered) model described by a system of ordinary differential equations. However, the model predictions vary according to the month in which the outbreak starts; the timing of the outbreak is randomly determined, and school attendance is eliminated for July and August. Each scenario is run over the course of five simulations, with the results being averaged and 95% confidence intervals being calculated via the standard deviation approach. The requisite number of simulations was determined by calculating the variance between model simulations, and running continuous simulations until the average value had a standard error below 5%. The model flow diagram is illustrated in Fig. 1.

From Fig. 1, we arrive at the system of ODEs presented below. Descriptions, ranges, and sample values for model parameters are provided in Table 6.

$$\begin{array}{cc} \frac{dS}{dt}=-\left(N_{C}+\beta \right)\cdot S & \text{Susceptible} \end{array}$$

$$\begin{array}{cc} \frac{dE}{dt}=\beta \cdot \left(S+\phi \cdot S_{V}\right)-E\cdot \varepsilon & \text{Latent}\;\text{Infected} \end{array}$$

$$\begin{array}{cc} \frac{dS_{V}}{dt}=\left(1-V_{e}\right)\cdot N_{C}\cdot S-\phi \cdot S_{V}\cdot \text{β} & \text{Susceptible}\;\text{with}\;\text{Failed}\;\text{Vaccination} \end{array}$$

$$\begin{array}{cc} \frac{dV}{dt}=V_{e}\cdot N_{C}\cdot S & \text{Vaccinated} \end{array}$$

$$\begin{array}{cc} \frac{dI_{C}}{dt}=\frac{2}{3}\cdot E\cdot \varepsilon -I_{C}\left(\theta +r+\zeta \right) & \text{Infected}\;\text{Symptomatic} \end{array}$$

$$\begin{array}{cc} \frac{dI_{A}}{dt}=\frac{1}{3}\cdot \left(E\right)\cdot \varepsilon -I_{A}\cdot r & \text{Infected}\;\text{Asymptomatic} \end{array}$$

$$\begin{array}{cc} \frac{dH}{dt}=\left(I_{C}\right)\cdot \theta -\left(r_{Hosp}+\chi_{Hosp}+\rho_{ICU}\right)\cdot H & \text{Hospitalized} \end{array}$$

$$\begin{array}{cc} \frac{dICU}{dt}=\rho_{ICU}\cdot H-\left(r_{ICU}+\chi_{ICU}\right)\cdot ICU & \text{Intensive}\;\text{Care}\;\text{Unit} \end{array}$$

**Table 6:** Model parameters.

Symbol	Definition	Sample value	References	Range
*N*_*C*_	Rate of vaccination	8.5e^−4^ (1/days)	(Sander, Bauch, et al., 2010)	8.5e^−4^ (1/days)
V_e_	Vaccine efficiency	65%	(Jefferson, Rivetti, Di Pietrantonj, Demicheli, & Ferroni, 2014)	40–90%
φ	Reduction in infectivity due to vaccination	35%	(Basta, Chao, Halloran, Matrajt, & Longini, 2009)	20–50%
ε	Rate of disease progression	1/1.6 days	(Andradottir et al., 2011)	1/3–1/7 (1/days)
θ	Rate of hospitalization	Age-dependent	(CIHI, 2010, Smetanin et al., 2009)	1e^−3^–1e^−1^ (1/days)
r	Rate of recovery	r = 1/4.8 Hosp = 1/3.35 ICU = 1/10.25	(Andradottir et al., 2011, Basta et al., 2009, Serres et al., 2010)	1/4–1/7 (1/days)
χ	Death rate in hospital setting	Hosp = 1e^−3^ ICU = 0.1	(Presannis et al., 2009)	1e^−3^–1e^−1^ (1/days)
ρ	Progression through hospital	ICU = 0.05	(Smetanin et al., 2009)	0.05–0.5

Each simulation begins with 50 infected cases being seeded across the five age groups in proportion to their relative size. A certain proportion (*N*_*C*_) of susceptible individuals can receive vaccination, moving either to an immune “vaccinated” group (V) or a less susceptible — relative to the unvaccinated group — “failed vaccinated” group (S_V_). Upon infection, individuals move to a latent, “exposed” group for a period of one to three days, followed by an infectious period of four to seven days (Serres et al., 2010, Tuite et al., 2010b). Two thirds will be symptomatic (I_C_), while the remaining third will be asymptomatic (I_A_) and half as infectious (Longini et al., 2004).

Of those who are symptomatic, a small proportion (0.4–1.0%) will require hospitalization for an average of four days (Baker et al., 2009, CIHI, 2010, Lum et al., 2009, Smetanin et al., 2009). Age-specific hospitalization rates were calibrated to reflect empirical data from the A(H1N1) pandemic using 2009–2010 data from the Influenza Hospitalization Surveillance Network (FluSurv-Net), available at (https://gis.cdc.gov/GRASP/Fluview/FluHospRates.html) (CDC, 2016). Acute-care patients will experience a 3.125% mortality rate, while an additional 16% will require ICU care and ventilation for an average of ten days, with an associated mortality rate of 50% (Presannis et al., 2009, Smetanin et al., 2009). Age-specific mortality rates given hospitalization are drawn from previously published Canadian studies (Sander, Bauch, et al., 2010). We assume that all deaths in those under 65 will occur in hospital, while 25% of deaths among seniors will occur in other settings, such as retirement homes and long-term care facilities. These estimates reflect empirical data from past pandemics in Canada. Model parameters are listed in Table 6.

### 2.4 Resources

Hospital-resource capacity was estimated using data from the Canadian Institute of Health Information (CIHI, 2016). Counts of “acute”, “ICU” and “other” beds were obtained for each hospital. Since Quebec hospitals do not report to CIHI, this information was obtained separately through the Quebec Ministry of Health and Social Services (SSSQ, 2016). As these hospitals did not report the number of ICU beds, we calculated the proportion of hospital beds outside of Quebec that were designated for the ICU (6.2%) and extrapolated this to Quebec hospitals. Hospitals were then mapped geographically using ArcGIS (ESRI, Ottawa, Canada), and hospitals within the borders of each CMA were combined for the purposes of the present analysis. Hospital-resource data were combined with Census demographic data to generated profiles for the 33 Canadian CMAs, included in Appendix A.

### 2.5 Vaccination

The vaccination strategy being modelled involves inter-wave vaccination — or “prevaccination” — of 25% of the general population. In other words, a quarter of the population will have received vaccination prior to the onset of the second pandemic wave. This coverage was selected on the basis of the lower bound of the 95% confidence interval from the lowest vaccine coverage reported in Canada between 2001 and 2012 (2010: 26.5%) (PHAC, 2014). We use a low coverage level to reflect a situation in which vaccine supply is limited, prioritizing high-risk populations. While recognizing that the first step of vaccine distribution could prioritize high-risk individuals, our interest is in identifying geographical areas where the hospital system is less able to accommodate surges in patient demand, and would therefore benefit from early vaccination to protect hospital-resource adequacy.

As informed by past empirical and modelling studies, we estimate vaccine efficacy for susceptibility (VE_S_) to be 65% (Jefferson et al., 2014, Saunders-Hastings et al., 2016). We also assume that vaccinated individuals who still become infected will be 35% less infectious (Basta et al., 2009) and 60% less likely to require hospitalization (McNeil et al., 2014, Skowronski et al., 2014). Effectiveness ranges were not incorporated in this study, as we seek to inform optimal vaccination strategies given a vaccine of a specified effectiveness.

### 2.6 Outcomes

Simulation results were calculated across six health outcomes of interest: cases of symptomatic infection, cases of hospitalization, cases of ICU admission, peak demand as a percentage of acute-care hospital capacity, peak ICU demand as a percentage of capacity and total deaths.

### 2.7 Analysis

Simulations for each of the 33 CMAs were run across eight vaccination–transmissibility–pathogenicity combinations. Summary measures for the six outcomes of interest are presented across all 264 scenarios. In this way, we conducted a univariate sensitivity analysis of the impact of shifting disease severity and population demographics.

We also associated the threat to health-system capacity with key demographic and hospital system characteristics, by plotting our results against the following CMA characteristics: proportion of total population that are seniors, adults, young adults, children and infants, as well as acute-care and ICU beds per 10,000 population. We assessed strength of linear relationships using the Pearson correlation coefficient (R), reported alongside the coefficient of determination (r^2^) in order to present both the strength and direction of relationships and the proportion of health outcome variance accounted for by these relationships. We used previously reported thresholds to qualify the strength of correlations, presented in Table 7.

**Table 7:** Interpretation of the size and strength of Pearson correlation coefficient (Hinkle & Jurs, 2003).

Correlation (R)	Interpretation
(±) 0.7–1.0	Strong correlation
(±) 0.5–0.69	Moderate correlation
(±) 0.3–0.49	Weak correlation
(±) 0–0.29	Negligible correlation

Sensitivity analyses for strength of association was undertaken using the five scenarios presented in Table 8. We evaluated the effect of increases in disease transmissibility and pathogenicity, vaccination and population susceptibility.

**Table 8:** Summary of five scenarios used for sensitivity analysis.

Scenario	Parameter			
	Transmissibility	Hospitalization rate (%)	Vaccination	Pre-existing immunity
1	0.275	0.4	No	Yes
2	0.3	0.4	No	Yes
3	0.275	1.0	No	Yes
4	0.275	0.4	Yes	Yes
5	0.275	0.4	No	No

In addition to the sensitivity analysis, this model has been validated through data parameterization, assessment of the structural validity and predictive validation (Carrasco et al., 2013). Parameterization was done by prioritizing empirical data from Canadian contexts to inform model inputs; structural validity was sought by grounding InFluNet in a solid understanding of epidemic theory and best practices informed by previously published modelling research (Andradottir et al., 2011, Del Valle et al., 2013, Del Valle et al., 2007, Gojovic et al., 2009, Sander et al., 2010a, Sander et al., 2010b); predictive validity was assessed by comparing simulated attack rates in a pandemic scenario similar to the 2009 H1N1 pandemic to empirical data (Tuite et al., 2010a, Tuite et al., 2010b) and a previously published modelling study of H1N1 transmission in a Canadian municipality (Andradottir et al., 2011). Similar attack rate estimates were understood to support the predictive validity of the model. REB approval was not required for this study, as all data are publically available and do not involve individual health information.

## 3 Results

The following subsections describe model findings as they relate to symptomatic cases, acute-care hospital admissions, ICU admissions, and mortality associated with a second pandemic wave. Each includes an analysis of how the predicted burden of the pandemic varies by disease characteristics and vaccination distribution, as well as an additional analysis of the demographic and health-system predictors of hospital-resource inadequacy. These subsections discuss major findings, while full summary tables of all simulations (Appendix B–G) and sensitivity analyses (Appendix H–I) are included in the supplementary material.

### 3.1 Symptomatic cases

As presented in Table 9, the average illness attack rate across the 33 CMAs was 23.7–37.2% for simulations with no vaccination; it varied between 2.4% and 6.5% for simulations with vaccination.

**Table 9:** Illness attack rate according to disease profile and vaccination status, averaged across 33 CMAs.

Disease profile	Vaccination status	Average illness attack rate (%)
R_0_ = 1.65; Hospitalization rate = 0.4%	No vaccination	23.7
	25% pre-vaccination	2.5
R_0_ = 1.80; Hospitalization rate = 0.4%	No vaccination	37.8
	25% pre-vaccination	6.5
R_0_ = 1.65; Hospitalization rate = 1.0%	No vaccination	23.2
	25% pre-vaccination	2.4
R_0_ = 1.80; Hospitalization rate = 1.0%	No vaccination	37.2
	25% pre-vaccination	6.4

Vaccination of 25% of the population prior to onset of the second pandemic wave reduced symptomatic cases by an average of 83.8%, with a minimum reduction of 73.0% (Peterborough, Ontario) and a maximum reduction of 96.6% (Toronto, Ontario). Vaccination became less effective in preventing symptomatic infection under scenarios with higher transmissibility (mean reduction of 70.0%, range 55.1–95.0%) but still resulted in a marked reduction in infection.

The relative representation of different age groups did not have a strong impact on the number of symptomatic cases across CMAs. Two mild predictors were the percentage of adults (R = 0.3185; r^2^ = 0.1014) and the percentage of seniors (R = −0.3774; r^2^ = 0.1424), which had opposite effects on cases of symptomatic infection. Increased disease transmissibility, vaccination and the removal of pre-existing immunity reduced this correlation, while increasing the hospitalization rate had no effect.

### 3.2 Hospitalizations

The total number of all acute-care hospital admissions across the 33 CMAs ranged from 18,884 to 62,168 in scenarios of no vaccination and from 790 to 4671 under scenarios where 25% of the population had been vaccinated. The CMA with the highest number of hospitalizations was Toronto, Ontario (2074–10,788 with no vaccination); the CMA with the lowest number of hospitalizations was Peterborough, Ontario (184–430 with no vaccination). However, the CMA with the highest proportion of hospitalizations was Brantford, Ontario (16.9–39.8 per 10,000 population with no vaccination); the lowest proportion was in Toronto, Ontario (3.7–19.3 per 10,000 population with no vaccination). The average number of hospitalizations across all CMAs ranged from 572 to 1884 (12.9–33.5 per 10,000 population) in scenarios with no intervention and from 24 to 142 (0.9–4.0 per 10,000) under scenarios when 25% of the population had been pre-vaccinated. Vaccination reduced the number of hospitalizations by an average of 88.6–93.8%, with lower impacts associated with higher disease transmissibility and not elevated pathogenicity.

The peak acute-care hospital demand — presented as a percentage of acute-care capacity — ranged from 7.5% to 19.5% in situations with no vaccination and from 0.6% to 2.6% in situations with 25% pre-vaccination. Vaccination reduced peak acute-care demand by an average of 86.3%–91.9%, with the greatest reductions in effectiveness seen in scenarios with higher assumed disease transmissibility. The eight CMAs at elevated risk of acute-care resource constraints — where baseline model assumptions resulted in peak acute-care demand in excess of 10% of capacity — are identified in Table 10. Seven of the eight are located in the Southern Ontario region and one was in British Columbia.

**Table 10:** CMAs at elevated risk of acute-care hospital-resource inadequacy. Figures presented are from model simulations reflecting a virus with an R_0_ of 1.65 and a hospitalization rate of 0.4%.

CMA	Peak range of acute care use as a percentage of total capacity [with no vaccination]	Peak range of acute care use as a percentage of total capacity [with 25% vaccination]
Brantford, Ontario	15.3–38.4	1.9–6.4
Oshawa, Ontario	15.2–38.2	1.1–5.7
Kitchener–Cambridge–Waterloo, Ontario	14.8–37.1	0.9–5.0
Guelph, Ontario	12.2–32.8	1.6–5.1
Saint Catharine's–Niagara, Ontario	12.8–32.1	0.9–4.6
Barrie, Ontario	12.2–30.5	1.3–5.1
Windsor, Ontario	11.5–29.0	0.9–4.5
Abbotsford–Mission, British Columbia	10.1–25.4	1.1–4.2

**Fig. 2:**
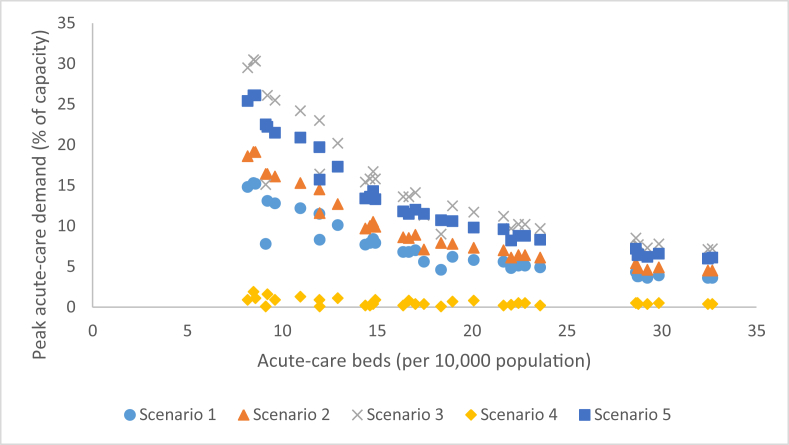
Peak acute-care demand as a function of acute-care beds staffed and in operation per 10,000 population across 33 CMAs and five sensitivity analysis scenarios. Scenario 1: R_0_ = 1.65, hospitalization rate = 0.4%; no intervention; pre-existing immunity in place; Scenario 2 R_0_ = 1.80, hospitalization rate = 0.4%; no intervention; pre-existing immunity in place; Scenario 3: R_0_ = 1.65, hospitalization rate = 1.0%; no intervention; pre-existing immunity in place; Scenario 4: R_0_ = 1.65, hospitalization rate = 0.4%; 25% pre-vaccination; pre-existing immunity in place; Scenario 5: R_0_ = 1.65, hospitalization rate = 0.4%; no intervention; no pre-existing immunity.

The proportion of the population comprised of children was a moderately strong demographic predictor examined in our assessment of peak acute-care demand (R = 0.6761; r^2^ = 0.4572). Increasing the hospitalization rate, vaccination coverage or population susceptibility weakened this correlation, while increasing disease transmissibility had no effect. A strong predictor was the number of acute-care beds per 10,000 population in each CMA (R = −0.8697; r^2^ = 0.7564). Increasing transmissibility or population susceptibility resulted in a stronger correlation; vaccination weakened the correlation and increased hospitalization rate had no effect. The association between acute-care hospital bed capacity and peak acute-care demand across the five scenarios is presented in Fig. 2.

### 3.3 ICU admissions

The total number of all ICU admissions across the 33 CMAs ranged from 4295 to 14,279 under scenarios of no vaccination and from 179 to 923 under scenarios with 25% pre-vaccination. As with hospitalizations, Toronto, Ontario, had the highest number of predicted ICU admissions (456–2437 with no vaccination) and Peterborough, Ontario, had the lowest (43–101 with no vaccination). However, the CMA with the highest proportion of ICU admission was Brantford, Ontario (3.8–8.9 per 10,000 population with no vaccination); the lowest proportion was in Toronto, Ontario (0.8–4.4 per 10,000 population with no vaccination). The average number of ICU admissions across all CMAs ranged from 130 to 433 (2.9–7.7 per 10,000 population) in scenarios with no vaccination and from 5 to 28 (0.2–0.9 per 10,000) when assuming 25% pre-vaccination. Vaccination reduced the number of ICU admissions by an average of 93.5–95.8%; increased disease transmissibility resulted in a small decrease in effectiveness, while increased pathogenicity had no effect.

The average peak ICU demand as a percentage of capacity ranged from 39.3% to 101.8% in situations with no vaccination and from 2.9% to 13.3% in situations with 25% pre-vaccination coverage. Vaccination reduced peak ICU demand by an average of 86.9–92.6%, with a small reduction in impact when disease transmissibility was increased. Of the 33 CMAs, 32 experienced a peak ICU demand above 10% of capacity under baseline assumption, while 18 experienced a peak ICU demand above 30%. These were identified as at elevated risk and are presented in Table 11: twelve CMAs are located in Ontario, four in British Columbia and two in Saskatchewan.

**Table 11:** CMAs at elevated risk of ICU-resource inadequacy. Figures presented are from model simulations reflecting a virus with an R_0_ of 1.65 and a hospitalization rate of 0.4%.

CMA	Peak range of ICU use as a percentage of total capacity [with no vaccination]	Peak range of ICU use as a percentage of total capacity [with 25% vaccination]
Saint Catharine's–Niagara, Ontario	97.9–243.4	5.8–32.0
Oshawa, Ontario	82.7–205.3	5.3–28.2
Abbotsford–Mission, British Columbia	79.1–205.3	8.1–33.0
Barrie, Ontario	70.1–174.0	6.8–28.9
Kitchener–Cambridge–Waterloo, Ontario	68.4–170.2	3.5–20.3
Brantford, Ontario	67.8–168.5	7.8–28.9
Victoria, British Columbia	62.0–154.5	4.0–21.2
Windsor, Ontario	54.6–135.5	3.8–19.4
Vancouver, British Columbia	52.7–170.9	0.9–6.5
Greater Sudbury, Ontario	50.0–124.4	5.3–21.1
Ottawa–Gatineau, Ontario-Quebec	49.8–131.3	1.2–8.3
Guelph, Ontario	45.2–112.3	5.1–19.2
Kelowna, British Columbia	41.7–103.8	4.2–17.3
Saskatoon, Saskatchewan	37.8–94.2	3.0–14.4
Peterborough, Ontario	35.2–87.6	4.3–15.1
Regina, Saskatchewan	35.1–87.2	3.2–14.1
Hamilton, Ontario	33.2–82.6	1.2–7.7
Thunder Bay, Ontario	31.3–77.7	3.8–13.4

**Fig. 3:**
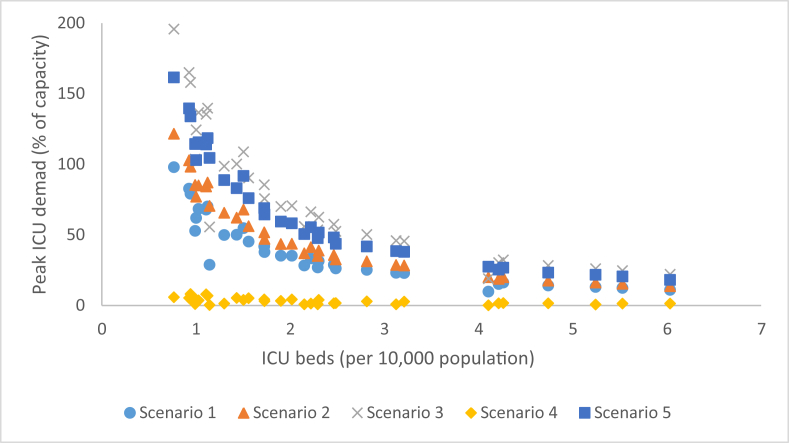
Peak ICU demand as a function of ICU beds staffed and in operation per 10,000 population across 33 CMAs and five sensitivity analysis scenarios. Scenario 1: R_0_ = 1.65, hospitalization rate = 0.4%; no intervention; pre-existing immunity in place; Scenario 2 R_0_ = 1.80, hospitalization rate = 0.4%; no intervention; pre-existing immunity in place; Scenario 3: R_0_ = 1.65, hospitalization rate = 1.0%; no intervention; pre-existing immunity in place; Scenario 4: R_0_ = 1.65, hospitalization rate = 0.4%; 25% pre-vaccination; pre-existing immunity in place; Scenario 5: R_0_ = 1.65, hospitalization rate = 0.4%; no intervention; no pre-existing immunity.

The proportion of the total population comprised of children was weakly correlated with peak ICU demand (R = 0.4955; r^2^ = 0.2456). Increased vaccination and population susceptibility were the only two parameters that affected correlation strength, with both parameters weakening the correlation. The strongest predictor was the number of ICU beds per 10,000 population (R = −0.8151; r^2^ = 0.6644). Increased disease transmissibility strengthened this correlation, while vaccination weakened it. Hospitalization rate and pre-existing immunity had no effect. The association between ICU bed capacity and peak ICU care demand across the five scenarios is presented in Fig. 3.

### 3.4 Mortality

Total mortality across the 33 CMAs ranged from 2258 to 7944 fatalities in scenarios with no intervention, and from 88 to 472 in scenarios with 25% pre-vaccination. Toronto, Ontario, had the highest estimated mortality (199–1130 with no vaccination) while Thunder Bay, Ontario, had the lowest (25–62 with no vaccination). Total and average mortality estimates are presented in Table 12.

**Table 12:** Total and average mortality, according to disease profile and vaccination status, across 33 CMAs.

Disease profile	Vaccination status	Mortality	
		Total	Average mortality per CMA
R_0_ = 1.65; Hospitalization rate = 0.4%	No vaccination	2258	68
	25% pre-vaccination	88	3
R_0_ = 1.80; Hospitalization rate = 0.4%	No vaccination	4003	121
	25% pre-vaccination	238	7
R_0_ = 1.65; Hospitalization rate = 1.0%	No vaccination	4423	134
	25% pre-vaccination	186	6
R_0_ = 1.80; Hospitalization rate = 1.0%	No vaccination	7944	241
	25% pre-vaccination	472	14

Pre-vaccination of 25% of the population reduced mortality by an average of 90.6–94.8%, with increased transmissibility resulting in a slight reduction in effect and increased pathogenicity having no effect. While there were no strong correlations between demographic profiles and mortality estimates, the two most notable correlations were mild associations with the population represented by infants (R = 0.3558; r^2^ = 0.1266) and by seniors (R = −0.4475; r^2^ = 0.2002). Both demonstrated stronger correlations under vaccination scenarios, weaker correlations under higher transmissibility and population susceptibility and were unaffected by increasing pathogenicity.

## 4 Discussion

To our knowledge, this is the first assessment of the preparedness of Canadian CMAs accommodate surges in patient demand during a second pandemic influenza wave with the help of inter-wave vaccination. The primary objective of this study was to evaluate the relative threat of a second-wave influenza pandemic across 33 Canadian CMAs. There were two secondary objectives: to evaluate the potential of inter-wave vaccination to contain a second pandemic wave, and to assess the strength of correlation of various demographic and health system characteristics in predicting pandemic burden and hospital-capacity adequacy. In this way, we sought to inform pandemic influenza vaccination planning in Canada and advance the identification of areas at high risk during influenza pandemics.

Canadian acute-care and ICU bed occupancy has been reported to routinely hover around 90% of total capacity (Frolic et al., 2009, Smetanin et al., 2009). Across 264 unique CMA–disease–intervention combinations, we found that, under scenarios of no intervention, eight of the 33 CMAs experienced a peak acute-care hospitalization rate above 10% under the most mild disease scenarios (R_0_ = 1.80; HR = 0.4%); seven of these were located in Southern Ontario. With respect to ICU demand, peak demand exceeded expected bed availability in all no-intervention scenarios except Montreal, Quebec, regardless of disease severity; the greatest strain is expected in Southern Ontario and British Columbia, while the Maritime, Prairie and Quebec CMAs appear to be at lower risk. From Fig. 2, Fig. 3, we suggest that the ability to accommodate surges in patient demand, even in the absence of vaccination, can be predicted by an acute-care and ICU-bed capacity threshold of 15/10,000 and 3/10,000, respectively. In summary, though patient demand for hospital beds may rarely exceed *total capacity*, in many cases likely bed *availability* was exceeded, suggesting that additional surge planning measures — such as triage and repurposing of beds — may be required.

Inter-wave vaccination was found to be very effective, suggesting that a combination of natural immunity from first-wave infection and conferred immunity from receipt of an effective vaccine may contribute to a substantial protective herd effect. Under scenarios with 25% pre-vaccination, no CMA experienced a peak acute-care hospital demand above 6.4% of capacity (Brantford, Ontario) under the most severe disease assumptions (R_0_ = 1.80; HR = 1.0%). While pre-vaccination was able to protect ICU-resource adequacy under mild disease assumptions, 7–8 CMAs experienced peak demand above 10% of capacity under moderate disease assumptions, and 16 exceeded 10% of capacity under severe disease assumptions. This points to the need for the early identification of novel viral strains, the rapid development and distribution of pandemic vaccines and targeting critical care surge planning, particularly in areas less able to accommodate surges in patient demand.

Demographic characteristics had some weak associations with predicted pandemic burden. These included risk increases associated with the proportion of adults and symptomatic cases (R = 0.3185, r^2^ = 0.1014), the proportion of infants and number of deaths (R = 0.3558, r^2^ = 0.1266) and the overall protective effect of a higher proportion of seniors. We found much stronger correlations between overall acute-care and ICU bed capacity and peak demand. This suggests that the supply-side, health system factors will be much more important in determining the ability to accommodate surges in patient demand than will characteristics of the community itself.

Our findings suggest that vaccine distribution strategies could benefit from prioritization of metropolitan areas with reduced acute-care and ICU-bed capacity. This research builds upon the existing research base in a coherent manner. A modelling study of Hamilton, Ontario, predicted an illness attack rate of 34.1% in the absence of any interventions (Andradottir et al., 2011); we predicted a similar rate of 31.5%. An assessment of pandemic vaccination in the United States mirrored our findings of a significantly protective effect of vaccination that decreased with increasing influenza transmissibility (Basta et al., 2009). Finally, another modelling study predicted Canadian ICU and ventilator shortages for attack rates above 20–25%, concluding that vaccination could significantly reduce ventilator demand (Smetanin et al., 2009). We extend this analysis by broadening the range of health-outcome measures, conducting metropolitan rather than provincial analyses and examining a second-wave pandemic when pandemic vaccination is realistically going to be available.

The present study is subject to certain limitations. First, we do not account for the protective effect of other interventions that could be implemented, including antiviral treatment and prophylaxis, voluntary isolation and personal protective measures. As a result, we have likely overestimated the actual burden that would arise from a second-wave pandemic; this was done in an effort to identify high-risk areas that would most benefit from early vaccine distribution, with all else being equal. Further, it should be noted that we assumed that a high-efficacy vaccine was available and would result in reductions to susceptibility, infectivity, hospitalization and, therefore, mortality.

Second, we do not consider the ethical implications of targeted resource allocation, as this was considered to be outside the scope of a paper focusing on the practical implications of vaccine distribution decisions. It must, however, be considered as planning for the next pandemic proceeds.

Third, our approach to estimating ICU capacity in Quebec as a proportion of total bed capacity may have overestimated critical-care capacity in the province. Indeed, peak acute-care demand was disproportionately higher in Quebec CMAs, relative to ICU demand, and more reliable estimates of capacity are needed. We also base our modelling assumptions related to movement through the hospital system on data from the mild 2009 H1N1; while lack of available data from earlier pandemics made this a necessary limitation, it may underestimate rates of critical illness and death given hospitalization.

Lastly, we treat each CMA as an independent unit, with no movement between areas; this may have overestimated the burden in smaller CMAs where infected individuals may seek care outside of their region. This possibility is particularly likely in the high-risk areas surrounding Toronto, where a higher density of critical care skills and resources results in referrals of complicated cases from surrounding areas. While this approach ignores the potential value of Local Health Integration Networks, it was chosen to allow assessment of individual CMA vulnerability. We also did not scale social contact rates according to variance in population density across CMAs, as this relationship remains poorly understood and difficult to quantify reliably (Hu, Nigmatulina, & Eckhoff, 2013). Instead, we included a transmission parameter based upon the proportion of infected individuals across the entire population, which would have the effect of decreasing transmission risk in high-population city centers similar to scaling contact rates. In addition, as contact rates saturate at higher population densities, our focus on urban areas should prevent undue bias from low population density (Hu et al., 2013).

Despite these limitations, the present study constitutes an informative evaluation of the differential preparedness of Canadian hospital systems to accommodate surges in patient demand during a second-wave influenza pandemic. We highlight high-risk areas in need of priority vaccine distribution, and suggest that supply-side health system profiles are the strongest determinant of pandemic vulnerability.

## 5 Conclusion

This study provides important insights into Canadian pandemic preparedness, employing the InFluNet model to evaluate potential burdens, assess preparedness, and identify predictive factors across the 33 Canadian Census Metropolitan Areas. Our analysis suggests that health systems in Southern Ontario and British Columbia are at greatest risk of being stressed by surges in patient demand, while areas in the Quebec, the Maritimes and Prairie provinces are better able to accommodate these increases. Assuming a high vaccine efficacy, inter-wave vaccination was found to be very effective in mitigating these threats, even under severe disease assumptions. Hospital capacity was a strong predictor of pandemic-associated pressures, while demographic characteristics had only mild correlations. Our study emphasizes the need for targeted early vaccine distribution to high-risk individuals and areas and points to the need for continued pandemic preparedness and surge capacity planning.

## Conflicts of interests

The authors declare that they have no conflicts of interest.

## References

[bib1] Achonu C., Rosella L., Gubbay J.B., Deeks S., Rebbapragada A., Mazzulli T., Crowcroft N.S. (2011). Seroprevalence of pandemic influenza H1N1 in Ontario from January 2009–May 2010. PLoS One.

[bib2] Andradottir S., Chiu W., Goldsman D., Lee M.L., Tsui K.L., Sander B., Nizam A. (2011). Reactive strategies for containing developing outbreaks of pandemic influenza. BMC Public Health.

[bib3] Baker M., Wilson N., Huang Q. (2009). Pandemic influenza A(H1N1) in New Zealand: The experience from April to August 2009. European Surveillance.

[bib4] Bansal S., Pourbohloul B., Hupert N., Grenfell B., Meyers L.A. (2010). The shifting demographic landscape of pandemic influenza. PLoS One.

[bib5] Basta N.E., Chao D.L., Halloran M.E., Matrajt L., Longini I.M. (2009). Strategies for pandemic and seasonal influenza vaccination of schoolchildren in the United States. American Journal of Epidemiology.

[bib6] Biggerstaff M., Cauchemez S., Reed C., Gambhir M., Finelli L. (2014). Estimates of the reproduction number for seasonal, pandemic, and zoonotic influenza: A systematic review of the literature. BMC Infectious Diseases.

[bib7] Carrasco L., Jit M., Chen M., Lee V., Milne G., Cook A. (2013). Trends in parameterization, economics and host behaviour in influenza pandemic modelling: A review and reporting protocol. Emerging Themes in Epidemiology.

[bib8] CDC (2016). Weekly U.S. Influenza surveillance report. http://www.cdc.gov/flu/weekly/.

[bib9] CIHI (2010). The impact of the H1N1 pandemic on Canadian hospitals. https://secure.cihi.ca/estore/productSeries.htm?pc=PCC545.

[bib10] CIHI (2016). Number of hospital beds staffed and in operation: Breakdown by grouped functional centre. https://www.cihi.ca/en/spending-and-health-workforce/spending/cmdb-hospital-beds-staffed-and-in-operation-2014-2015.

[bib11] Del Valle S.Y., Hyman J., Chitnis N. (2013). Mathematical models of contact patterns between age groups for predicting the spread of infectious diseases. Mathematical Biosciences and Engineering.

[bib12] Del Valle S.Y., Hyman J.M., Hethcote H.W., Eubank S.G. (2007). Mixing patterns between age groups in social networks. Social Networks.

[bib13] van den Driessche P., Watmough J. (2002). Reproduction numbers and sub-threshold endemic equilibria for compartmental models of disease transmission. Mathematical Biosciences.

[bib14] Fiore A., Shay D., Broder K., Iskander J., Uyeki T., Mootrey G., Cox N. (2008). Prevention and control of influenza: Recommendations of the advisory committee on immunization practices (ACIP), 2008. Morbidity and Mortality Weekly Report.

[bib15] Frolic A., Kata A., Kraus P. (2009). Development of a critical care triage protocol for pandemic influenza: Integrating ethics, evidence, and effectiveness. Healthcare Quarterly.

[bib16] Gojovic M.Z., Sande B., Fisman D., Krahn M.D., Bauch C.T. (2009). Modeling mitigation strategies for pandemic (H1N1). CMAJ.

[bib17] Hinkle D.E., Jurs S.G. (2003). Applied Statistics for the behavioural Sciences.

[bib18] Hu H., Nigmatulina K., Eckhoff P. (2013). The scaling of contact rates with population density for the infectious disease models. Mathematical Biosciences.

[bib19] Jefferson T., Rivetti A., Di Pietrantonj C., Demicheli V., Ferroni E. (2014). Vaccines for preventing influenza in healthy children. Cochrane Database of Systematic Reviews.

[bib20] Longini I., Halloran M.E., Nizam A., Yang Y. (2004). Containing pandemic influenza with antiviral agents. American Journal of Epidemiology.

[bib21] Lum M., McMillan A., Brook C., Lester R., Piers L. (2009). Impact of pandemic (H1N1) 2009 influenza on critical care capacity in Victoria. Medical Journal of Australia.

[bib22] McNeil S., Shinde V., Andrew M., Hatchette T., Leblanc J., Ambrose A., McGeer A. (2014). Interim estimates of 2013/14 influenza clinical severity and vaccine effectiveness in the prevention of laboratory-confirmed influenza-related hospitalisation, Canada, February 2014. Euro Surveillance.

[bib23] Oshitani H., Kamigaki T., Suzuki A. (2008). Major issues and challenges of influenza pandemic preparedness in developing countries. Emerging Infectious Diseases.

[bib24] PHAC (2014). Vaccine coverage amongst adult Canadians: Results from the 2012 adult national immunization coverage (aNIC) survey. http://www.phac-aspc.gc.ca/im/nics-enva/vcac-cvac-eng.php.

[bib25] Presannis A.M., Angelis D., Hagy A., Reed C., Riley S., Lipsitch M., The New York Swine Flu Investigation Team (2009). The severity of pandemic H1N1 influenza in the United States, from April to July 2009: A bayesian analysis. PLos Medicine.

[bib26] Reed C., Katz J.M., Hancock K., Balish A., Fry A.M., Group H.N.S.W. (2012). Prevalence of seropositivity to pandemic influenza A/H1N1 virus in the United States following the 2009 pandemic. PLoS One.

[bib27] Sander B., Bauch C.T., Fisman D., Fowler R.A., Kwong J.C., Maetzel A., Krahn M. (2010). Is a mass immunization program for pandemic (H1N1) 2009 good value for money? Evidence from the Canadian experience. Vaccine.

[bib28] Sander B., Kwing J.C., Bauch C.T., Maetzel A., McGeer A., Raboud J.M. (2010). Economic appraisal of Ontario's universal influenza immunization program: Cost-utility analysis. PLoS Medicine.

[bib29] Saunders-Hastings P., Krewski D. (2016). Reviewing the history of pandemic influenza: Understanding patterns of emergence and transmission. Pathogens.

[bib30] Saunders-Hastings P., Reisman J., Krewski D. (2016). Assessing the state of knowledge regarding the effectiveness of interventions to contain pandemic influenza transmission: A systematic review and narrative synthesis. PLoS One.

[bib31] Serres G., Rouleau I., Hamelin M., Quach C., Skowronski D.M., Flamand L., Boivin G. (2010). Contagious period for pandemic (H1N1) 2009. Emerging Infectious Diseases.

[bib32] Skowronski D., Chambers C., Sabaiduc S., De Serres G., Dickinson J., Winter A., Li Y. (2014). Interim estimates of 2013/14 vaccine effectiveness against influenza A(H1N1)pdm09 from Canada s sentinel surveillance network, January 2014. Euro Surveillance.

[bib33] Smetanin P., Stiff D., Kumar A., Kobak P., Zarychanski M., Simonsen M. (2009). Potential intensive care unit ventilator demand- capacity mismatch due to novel swine-origin H1N1 in Canada. Canadian Journal of Infectious Diseases and Medical Microbiology.

[bib34] SSSQ (2016). Institutions: Legal entities. http://wpp01.msss.gouv.qc.ca/appl/M02/M02ListeEtab.asp.

[bib35] StatsCan (2010). General social survey - 2010 overview of the time use of Canadians.

[bib36] Tuite A.R., Fisman D.N., Kwong J.C., Greer A.L. (2010). Optimal pandemic influenza vaccine allocation strategies for the Canadian population. PLoS One.

[bib37] Tuite A.R., Greer A.L., Whelan M., Winter A.L., Lee B., Yan P., Fisman D.N. (2010). Estimated epidemiologic parameters and morbidity associated with pandemic H1N1 influenza. Canadian Medical Association Journal.

[bib38] Yang Y., Sugimoto J.D., Halloran M.E., Basta N.E., Chao D.L., Matrajt L., Longini I.M. (2009). The transmissibility and control of pandemic influenza A (H1N1) virus. Science.

