# Supplementary material for: National assessment of Canadian pandemic preparedness: Employing InFluNet to identify high-risk areas for inter-wave vaccine distribution
Source: Infect Dis Model. 2017 Jul 5;2(3):341–52. doi: 10.1016/j.idm.2017.06.005 (PMC6002068; doi:10.1016/j.idm.2017.06.005)
Supplement: Supplementary file 1 [file mmc1.docx]

**Appendix A. Census Metropolitan Area profiles**

| **Census Metropolitan Area** | **Province** | **Population by Age** | | | | | | **Hospital resources** | | |
| --- | --- | --- | --- | --- | --- | --- | --- | --- | --- | --- |
|  |  | **0 to 4** | **5 to 18** | **19 to 29** | **30 to 64** | **65+** | **Total** | **Acute beds** | **ICU beds** | **Total beds** |
| St. John's | NL | 10,725 | 29,275 | 32,385 | 99,530 | 25,055 | 196,970 | 576 | 83 | 1,022 |
| Halifax | NS | 19,965 | 58,125 | 65,070 | 196,060 | 51,105 | 390,325 | 861 | 97 | 1,565 |
| Moncton | NB | 7,410 | 22,195 | 19,090 | 69,675 | 20,265 | 138,635 | 453 | 39 | 705 |
| Saint John | NB | 6,740 | 21,045 | 16,880 | 63,780 | 19,315 | 127,760 | 366 | 41 | 640 |
| Saguenay | QC | 7,735 | 22,285 | 21,050 | 79,175 | 27,540 | 157,785 | 454 | 75 | 1,206 |
| Sherbrooke | QC | 10,860 | 30,825 | 30,845 | 95,715 | 33,640 | 201,885 | 655 | 112 | 1,800 |
| Trois-Rivieres | QC | 7,175 | 20,680 | 21,085 | 73,450 | 29,385 | 151,775 | 436 | 92 | 1,477 |
| Montreal | QC | 222,225 | 601,070 | 558,885 | 1,882,195 | 559,845 | 3,824,220 | 7,023 | 1,570 | 25,324 |
| Quebec City | QC | 40,775 | 115,790 | 103,515 | 379,410 | 126,235 | 765,725 | 1,807 | 401 | 6,474 |
| Ottawa-Gatineau | ON-QC | 71,245 | 204,920 | 190,640 | 622,640 | 156,875 | 1,246,320 | 1,822 | 162 | 4,336 |
| Kingston | ON | 7,865 | 24,080 | 26,010 | 75,625 | 25,975 | 159,555 | 266 | 68 | 880 |
| Peterborough | ON | 5,665 | 17,710 | 17,645 | 54,785 | 23,165 | 118,970 | 239 | 24 | 392 |
| Oshawa | ON | 20,965 | 66,845 | 48,830 | 174,910 | 44,625 | 356,175 | 306 | 33 | 914 |
| Toronto | ON | 318,900 | 953,325 | 844,425 | 2,759,750 | 706,665 | 5,583,065 | 5,094 | 638 | 11,074 |
| Hamilton | ON | 38,350 | 120,505 | 101,820 | 344,810 | 115,565 | 721,050 | 1,067 | 160 | 2,241 |
| St. Catherine’s-Niagara | ON | 18,365 | 61,335 | 51,600 | 185,750 | 75,125 | 392,175 | 377 | 30 | 874 |
| Windsor | ON | 17,330 | 61,055 | 39,105 | 154,170 | 47,585 | 319,245 | 382 | 48 | 860 |
| Kitchener-Cambridge-Waterloo | ON | 28,790 | 82,960 | 76,615 | 229,335 | 59,455 | 477,155 | 390 | 49 | 819 |
| Brantford | ON | 7,950 | 24,060 | 18,255 | 64,625 | 20,615 | 135,505 | 115 | 15 | 262 |
| Guelph | ON | 8,195 | 23,885 | 22,200 | 67,835 | 18,980 | 141,095 | 130 | 22 | 569 |
| London | ON | 26,150 | 78,245 | 73,950 | 225,340 | 71,100 | 474,785 | 808 | 117 | 1,694 |
| Barrie | ON | 10,795 | 35,990 | 25,920 | 90,790 | 23,525 | 187,020 | 205 | 21 | 325 |
| Greater Sudbury | ON | 8,080 | 25,290 | 22,535 | 79,025 | 25,835 | 160,765 | 305 | 23 | 502 |
| Thunder Bay | ON | 5,660 | 18,500 | 17,025 | 59,525 | 20,880 | 121,590 | 363 | 28 | 526 |
| Winnipeg | MB | 40,550 | 123,550 | 113,135 | 349,930 | 102,850 | 730,015 | 1,582 | 228 | 2,757 |
| Regina | SK | 13,225 | 34,855 | 35,630 | 99,225 | 27,625 | 210,560 | 480 | 40 | 720 |
| Saskatoon | SK | 16,625 | 43,545 | 49,545 | 119,300 | 31,585 | 260,600 | 585 | 45 | 853 |
| Calgary | AL | 80,855 | 201,450 | 200,125 | 613,670 | 118,750 | 1,214,850 | 1,747 | 261 | 2,782 |
| Edmonton | AL | 73,645 | 189,575 | 203,075 | 561,330 | 132,250 | 1,159,875 | 1,900 | 266 | 3,429 |
| Kelowna | BC | 8,305 | 26,805 | 25,015 | 85,270 | 34,450 | 179,845 | 268 | 31 | 458 |
| Abbotsford-Mission | BC | 10,705 | 31,400 | 25,205 | 78,835 | 24,040 | 170,185 | 220 | 16 | 793 |
| Vancouver | BC | 115,185 | 354,565 | 359,040 | 1,171,635 | 312,905 | 2,313,330 | 2,770 | 229 | 6,639 |
| Victoria | BC | 14,775 | 45,330 | 74,720 | 151,445 | 63,435 | 349,705 | 611 | 35 | 1,670 |
| Total | CA | 1,301,785 | 3,771,070 | 3,530,870 | 11,358,545 | 3,176,245 | 23,138,515 | 34,663 | 5,098 | 86,582 |
| Average | CA | 39,448 | 114,275 | 106,996 | 344,198 | 96,250 | 701,167 | 1,050 | 154 | 2,624 |
| Total Canada | CA | 1,877,095 | 5,460,255 | 4,805,170 | 16,389,110 | 4,945,065 | 33,476,695 | 54,746 | 7,604 | 139,299 |
| % Population Covered by CMA | CA | 69.4 | 69.1 | 73.5 | 69.3 | 64.2 | 69.1 | 63.3 | 67.0 | 62.2 |

**Appendix B. Symptomatic cases by CMA and disease scenario.**

Results presented reflect the average value across five simulations. Confidence intervals were not included due to a very small degree of variance across simulations, which suggests that the timing of an outbreak — as it relates to school terms — has only a small impact on overall pandemic burden.

| **Census Metropolitan Area** | **Scenario** | | | | | | | | | | | |
| --- | --- | --- | --- | --- | --- | --- | --- | --- | --- | --- | --- | --- |
|  | **Ro=1.65; HR=0.4%*** | | | **Ro=1.80; HR=0.4%%** | | | **Ro=1.65; HR=1.0%** | | | **Ro=1.80; HR=1.0%** | | |
|  | **No Vaccination** | **25% Pre-vaccination** | **Reduction (%)** | **No Vaccination** | **25% Pre-vaccination** | **Reduction (%)** | **No Vaccination** | **25% Pre-vaccination** | **Reduction (%)** | **No Vaccination** | **25% Pre-vaccination** | **Reduction (%)** |
| St. John's | 77,880 | 15,849 | 79.6% | 91,778 | 34,755 | 62.1% | 77,213 | 15,633 | 79.8% | 91,351 | 34,454 | 62.3% |
| Halifax | 141,322 | 18,755 | 86.7% | 177,135 | 49,308 | 72.2% | 139,667 | 18,464 | 86.8% | 176,136 | 48,716 | 72.3% |
| Moncton | 56,287 | 14,059 | 75.0% | 64,989 | 27,897 | 57.1% | 55,831 | 13,872 | 75.2% | 64,684 | 27,659 | 57.2% |
| Saint John | 52,080 | 13,572 | 73.9% | 59,903 | 26,333 | 56.0% | 51,658 | 13,391 | 74.1% | 59,618 | 26,110 | 56.2% |
| Saguenay | 63,985 | 14,828 | 76.8% | 74,294 | 30,539 | 58.9% | 63,509 | 14,648 | 76.9% | 71,982 | 30,295 | 57.9% |
| Sherbrooke | 79,304 | 15,869 | 80.0% | 93,686 | 35,089 | 62.5% | 78,595 | 15,646 | 80.1% | 93,233 | 34,751 | 62.7% |
| Trois-Rivieres | 61,758 | 14,728 | 76.2% | 71,544 | 29,936 | 58.2% | 61,321 | 14,556 | 76.3% | 71,266 | 29,710 | 58.3% |
| Montreal | 581,001 | 22,432 | 96.1% | 1,261,059 | 80,047 | 93.7% | 560,902 | 22,003 | 96.1% | 1,238,751 | 78,531 | 93.7% |
| Quebec City | 239,994 | 20,667 | 91.4% | 332,432 | 62,507 | 81.2% | 235,979 | 20,314 | 91.4% | 329,964 | 61,585 | 81.3% |
| Ottawa-Gatineau | 331,460 | 21,323 | 93.6% | 510,590 | 69,360 | 86.4% | 323,765 | 20,913 | 93.5% | 505,680 | 68,139 | 86.5% |
| Kingston | 64,080 | 14,690 | 77.1% | 74,568 | 30,406 | 59.2% | 63,565 | 14,500 | 77.2% | 74,229 | 30,145 | 59.4% |
| Peterborough | 48,864 | 13,203 | 73.0% | 56,002 | 25,118 | 55.1% | 48,514 | 13,047 | 73.1% | 55,764 | 34,932 | 37.4% |
| Oshawa | 128,408 | 17,978 | 86.0% | 159,902 | 46,311 | 71.0% | 126,572 | 17,632 | 86.1% | 158,781 | 45,630 | 71.3% |
| Toronto | 649,859 | 22,340 | 96.6% | 1,615,869 | 81,211 | 95.0% | 622,822 | 21,933 | 96.5% | 1,557,957 | 79,508 | 94.9% |
| Hamilton | 227,092 | 20,251 | 91.1% | 311,977 | 60,659 | 80.6% | 222,996 | 19,871 | 91.1% | 309,460 | 59,674 | 80.7% |
| St. Catherine’s-Niagara | 141,360 | 18,679 | 86.8% | 177,412 | 49,185 | 72.3% | 139,610 | 18,374 | 86.8% | 176,353 | 48,562 | 72.5% |
| Windsor | 116,900 | 17,552 | 85.0% | 143,927 | 43,986 | 69.4% | 115,281 | 17,213 | 85.1% | 142,931 | 43,351 | 69.7% |
| Kitchener-Cambridge-Waterloo | 164,047 | 19,018 | 88.4% | 211,564 | 52,462 | 75.2% | 161,544 | 18,664 | 88.4% | 210,051 | 51,676 | 75.4% |
| Brantford | 54,532 | 13,724 | 74.8% | 62,996 | 27,111 | 57.0% | 54,043 | 13,523 | 75.0% | 62,667 | 26,857 | 57.1% |
| Guelph | 56,759 | 13,885 | 75.5% | 65,684 | 27,795 | 57.7% | 56,268 | 13,688 | 75.7% | 65,355 | 27,541 | 57.9% |
| London | 164,212 | 19,124 | 88.4% | 211,408 | 52,672 | 75.1% | 161,849 | 18,786 | 88.4% | 209,982 | 51,925 | 75.3% |
| Barrie | 72,825 | 15,167 | 79.2% | 85,811 | 32,899 | 61.7% | 72,022 | 14,904 | 79.3% | 85,291 | 32,512 | 61.9% |
| Greater Sudbury | 64,541 | 14,750 | 77.1% | 75,125 | 30,582 | 59.3% | 63,999 | 14,549 | 77.3% | 74,769 | 30,308 | 59.5% |
| Thunder Bay | 49,969 | 13,377 | 73.2% | 57,301 | 25,585 | 55.3% | 49,603 | 13,215 | 73.4% | 57,053 | 25,390 | 55.5% |
| Winnipeg | 228,614 | 20,214 | 91.2% | 314,954 | 60,684 | 80.7% | 224,410 | 19,830 | 91.2% | 312,365 | 59,693 | 80.9% |
| Regina | 81,673 | 15,883 | 80.6% | 96,922 | 35,579 | 63.3% | 80,864 | 15,638 | 80.7% | 96,405 | 35,202 | 63.5% |
| Saskatoon | 98,314 | 16,748 | 83.0% | 118,758 | 39,801 | 66.5% | 97,232 | 16,476 | 83.1% | 118,071 | 39,336 | 66.7% |
| Calgary | 325,256 | 21,214 | 93.5% | 498,553 | 68,793 | 86.2% | 317,730 | 20,804 | 93.5% | 493,486 | 67,577 | 86.3% |
| Edmonton | 315,223 | 21,085 | 93.3% | 478,096 | 67,978 | 85.8% | 308,184 | 20,684 | 93.3% | 473,405 | 66,802 | 85.9% |
| Kelowna | 71,679 | 15,375 | 78.6% | 83,975 | 32,891 | 60.8% | 71,084 | 15,170 | 78.7% | 83,589 | 32,597 | 61.0% |
| Abbotsford-Mission | 66,845 | 14,684 | 78.0% | 78,300 | 31,027 | 60.4% | 66,163 | 14,445 | 78.2% | 77,853 | 30,691 | 60.6% |
| Vancouver | 472,259 | 22,231 | 95.3% | 864,668 | 76,882 | 91.1% | 458,898 | 21,822 | 95.2% | 853,350 | 75,516 | 91.2% |
| Victoria | 128,820 | 18,295 | 85.8% | 159,565 | 46,850 | 70.6% | 127,572 | 18,051 | 85.9% | 158,506 | 46,376 | 70.7% |

*R_0_ = basic reproductive rate; HR = hospitalization rate (as a percentage of symptomatic cases)

**Appendix C. Cases of acute-care hospital admission, by CMA and disease scenario.**

Results presented reflect the average value across five simulations. Confidence intervals were not included due to a very small degree of variance across simulations, which suggests that the timing of an outbreak — as it relates to school terms — has only a small impact on overall pandemic burden. Table C.1 presents percent reductions as a result of inter-wave vaccination, by CMA and disease scenario. Table C.2 presents hospitalization proportions, by CMA and disease scenario.

**Table C.1** Predicted number of hospitalizations by CMA, disease scenario and vaccination status.

| **Census Metropolitan Area** | **Scenario** | | | | | | | | | | | |
| --- | --- | --- | --- | --- | --- | --- | --- | --- | --- | --- | --- | --- |
|  | **Ro=1.65; HR=0.4%*** | | | **Ro=1.80; HR=0.4%%** | | | **Ro=1.65; HR=1.0%** | | | **Ro=1.80; HR=1.0%** | | |
|  | **No Vaccination** | **25% Pre-vaccination** | **Reduction (%)** | **No Vaccination** | **25% Pre-vaccination** | **Reduction (%)** | **No Vaccination** | **25% Pre-vaccination** | **Reduction (%)** | **No Vaccination** | **25% Pre-vaccination** | **Reduction (%)** |
| St. John's | 261 | 20 | 92.3% | 315 | 44 | 86.0% | 523 | 40 | 92.4% | 633 | 89 | 85.9% |
| Halifax | 464 | 23 | 95.0% | 604 | 61 | 89.9% | 925 | 46 | 95.0% | 1,213 | 122 | 89.9% |
| Moncton | 212 | 20 | 90.6% | 249 | 40 | 83.9% | 425 | 40 | 90.6% | 501 | 81 | 83.8% |
| Saint John | 204 | 20 | 90.2% | 238 | 40 | 83.2% | 408 | 40 | 90.2% | 479 | 79 | 83.5% |
| Saguenay | 223 | 19 | 91.5% | 264 | 40 | 84.8% | 446 | 38 | 91.5% | 530 | 81 | 84.7% |
| Sherbrooke | 289 | 22 | 92.4% | 350 | 49 | 86.0% | 579 | 43 | 92.6% | 703 | 97 | 86.2% |
| Trois-Rivieres | 213 | 19 | 91.1% | 251 | 39 | 84.5% | 426 | 38 | 91.1% | 506 | 79 | 84.4% |
| Montreal | 1,801 | 29 | 98.4% | 4,199 | 100 | 97.6% | 3512 | 58 | 98.3% | 8,314 | 800 | 90.4% |
| Quebec City | 818 | 27 | 96.7% | 1,200 | 81 | 93.3% | 1,623 | 54 | 96.7% | 2,402 | 161 | 93.3% |
| Ottawa-Gatineau | 1,115 | 28 | 97.5% | 1,837 | 90 | 95.1% | 2,195 | 56 | 97.4% | 3,665 | 179 | 95.1% |
| Kingston | 231 | 20 | 91.3% | 274 | 42 | 84.7% | 462 | 39 | 91.6% | 550 | 84 | 84.7% |
| Peterborough | 184 | 19 | 89.7% | 214 | 36 | 83.2% | 368 | 37 | 89.9% | 430 | 73 | 83.0% |
| Oshawa | 527 | 28 | 94.7% | 680 | 72 | 89.4% | 1,047 | 55 | 94.7% | 1,360 | 143 | 89.5% |
| Toronto | 2,074 | 31 | 98.5% | 5,482 | 107 | 98.0% | 4,015 | 61 | 98.5% | 10,788 | 212 | 98.0% |
| Hamilton | 846 | 29 | 96.6% | 1,227 | 86 | 93.0% | 1,674 | 58 | 96.5% | 2,453 | 171 | 93.0% |
| St. Catharine’s-Niagara | 535 | 27 | 95.0% | 698 | 70 | 90.0% | 1,065 | 53 | 95.0% | 1,400 | 140 | 90.0% |
| Windsor | 511 | 29 | 94.3% | 649 | 73 | 88.8% | 1,014 | 57 | 94.4% | 1,298 | 145 | 88.8% |
| Kitchener-Cambridge-Waterloo | 612 | 27 | 95.6% | 824 | 74 | 91.0% | 1,215 | 53 | 95.6% | 1,649 | 147 | 91.1% |
| Brantford | 229 | 22 | 90.4% | 269 | 44 | 83.6% | 457 | 43 | 90.6% | 539 | 87 | 83.9% |
| Guelph | 220 | 20 | 90.9% | 259 | 41 | 84.2% | 440 | 40 | 90.9% | 519 | 83 | 84.0% |
| London | 606 | 27 | 95.5% | 814 | 73 | 91.0% | 1,203 | 53 | 95.6% | 1,630 | 146 | 91.0% |
| Barrie | 313 | 24 | 92.3% | 377 | 54 | 85.7% | 624 | 49 | 92.1% | 755 | 108 | 85.7% |
| Greater Sudbury | 243 | 21 | 91.4% | 289 | 44 | 84.8% | 486 | 41 | 91.6% | 580 | 88 | 84.8% |
| Thunder Bay | 187 | 19 | 89.8% | 217 | 37 | 82.9% | 374 | 37 | 90.1% | 436 | 74 | 83.0% |
| Winnipeg | 834 | 28 | 96.6% | 1,212 | 84 | 93.1% | 1,649 | 56 | 96.6% | 2,423 | 167 | 93.1% |
| Regina | 303 | 22 | 92.7% | 369 | 50 | 86.4% | 606 | 44 | 92.7% | 740 | 100 | 86.5% |
| Saskatoon | 357 | 23 | 93.6% | 443 | 55 | 87.6% | 712 | 45 | 93.7% | 889 | 109 | 87.7% |
| Calgary | 1,055 | 27 | 97.4% | 1,727 | 86 | 95.0% | 2,079 | 54 | 97.4% | 3,448 | 171 | 95.0% |
| Edmonton | 1,031 | 27 | 97.4% | 1,669 | 86 | 94.8% | 2,034 | 54 | 97.3% | 3,333 | 170 | 94.9% |
| Kelowna | 267 | 21 | 92.1% | 319 | 46 | 85.6% | 533 | 43 | 91.9% | 641 | 93 | 85.5% |
| Abbotsford-Mission | 283 | 23 | 91.9% | 338 | 50 | 85.2% | 564 | 46 | 91.8% | 677 | 100 | 85.2% |
| Vancouver | 1,444 | 28 | 98.1% | 2,850 | 93 | 96.7% | 283 | 55 | 80.6% | 5,672 | 185 | 96.7% |
| Victoria | 392 | 21 | 94.6% | 503 | 54 | 89.3% | 784 | 42 | 94.6% | 1,012 | 107 | 89.4% |

*R_0_ = basic reproductive rate; HR = hospitalization rate (as a percentage of symptomatic cases)

**Table C.2** Predicted proportion of hospitalizations (per 10,000 population) by CMA, disease scenario and vaccination status.

| **Census Metropolitan Area** | **Scenario** | | | | | | | |
| --- | --- | --- | --- | --- | --- | --- | --- | --- |
|  | **Ro - 1.65; HR = 0.4%** | | **Ro=1.80; HR=0.4%** | | **Ro=1.65; HR=1.0%** | | **Ro=1.80; HR=1.0%** | |
|  | **No vaccination** | **25% Pre-vaccination** | **No vaccination** | **25% Pre-vaccination** | **No vaccination** | **25% Pre-vaccination** | **No vaccination** | **25% Pre-vaccination** |
| St. John's | 13.3 | 1.0 | 16.0 | 2.2 | 26.6 | 2.0 | 32.1 | 4.5 |
| Halifax | 11.9 | 0.6 | 15.5 | 1.6 | 23.7 | 1.2 | 31.1 | 3.1 |
| Moncton | 15.3 | 1.4 | 18.0 | 2.9 | 30.7 | 2.9 | 36.1 | 5.8 |
| Saint John | 16.0 | 1.6 | 18.6 | 3.1 | 31.9 | 3.1 | 37.5 | 6.2 |
| Saguenay | 14.1 | 1.2 | 16.7 | 2.5 | 28.3 | 2.4 | 33.6 | 5.1 |
| Sherbrooke | 14.3 | 1.1 | 17.3 | 2.4 | 28.7 | 2.1 | 34.8 | 4.8 |
| Trois-Rivieres | 14.0 | 1.3 | 16.5 | 2.6 | 28.1 | 2.5 | 33.3 | 5.2 |
| Montreal | 4.7 | 0.1 | 11.0 | 0.3 | 9.2 | 0.2 | 21.7 | 2.1 |
| Quebec City | 10.7 | 0.4 | 15.7 | 1.1 | 21.2 | 0.7 | 31.4 | 2.1 |
| Ottawa-Gatineau | 8.9 | 0.2 | 14.7 | 0.7 | 17.6 | 0.4 | 29.4 | 1.4 |
| Kingston | 14.5 | 1.3 | 17.2 | 2.6 | 29.0 | 2.4 | 34.5 | 5.3 |
| Peterborough | 15.5 | 1.6 | 18.0 | 3.0 | 30.9 | 3.1 | 36.1 | 6.1 |
| Oshawa | 14.8 | 0.8 | 19.1 | 2.0 | 29.4 | 1.5 | 38.2 | 4.0 |
| Toronto | 3.7 | 0.1 | 9.8 | 0.2 | 7.2 | 0.1 | 19.3 | 0.4 |
| Hamilton | 11.7 | 0.4 | 17.0 | 1.2 | 23.2 | 0.8 | 34.0 | 2.4 |
| St. Catharines-Niagara | 13.6 | 0.7 | 17.8 | 1.8 | 27.2 | 1.4 | 35.7 | 3.6 |
| Windsor | 16.0 | 0.9 | 20.3 | 2.3 | 31.8 | 1.8 | 40.7 | 4.5 |
| Kitchener-Cambridge-Waterloo | 12.8 | 0.6 | 17.3 | 1.6 | 25.5 | 1.1 | 34.6 | 3.1 |
| Brantford | 16.9 | 1.6 | 19.9 | 3.2 | 33.7 | 3.2 | 39.8 | 6.4 |
| Guelph | 15.6 | 1.4 | 18.4 | 2.9 | 31.2 | 2.8 | 36.8 | 5.9 |
| London | 12.8 | 0.6 | 17.1 | 1.5 | 25.3 | 1.1 | 34.3 | 3.1 |
| Barrie | 16.7 | 1.3 | 20.2 | 2.9 | 33.4 | 2.6 | 40.4 | 5.8 |
| Greater Sudbury | 15.1 | 1.3 | 18.0 | 2.7 | 30.2 | 2.6 | 36.1 | 5.5 |
| Thunder Bay | 15.4 | 1.6 | 17.8 | 3.0 | 30.8 | 3.0 | 35.9 | 6.1 |
| Winnipeg | 11.4 | 0.4 | 16.6 | 1.2 | 22.6 | 0.8 | 33.2 | 2.3 |
| Regina | 14.4 | 1.0 | 17.5 | 2.4 | 28.8 | 2.1 | 35.1 | 4.7 |
| Saskatoon | 13.7 | 0.9 | 17.0 | 2.1 | 27.3 | 1.7 | 34.1 | 4.2 |
| Calgary | 8.7 | 0.2 | 14.2 | 0.7 | 17.1 | 0.4 | 28.4 | 1.4 |
| Edmonton | 8.9 | 0.2 | 14.4 | 0.7 | 17.5 | 0.5 | 28.7 | 1.5 |
| Kelowna | 14.8 | 1.2 | 17.7 | 2.6 | 29.6 | 2.4 | 35.6 | 5.2 |
| Abbotsford-Mission | 16.6 | 1.4 | 19.9 | 2.9 | 33.1 | 2.7 | 39.8 | 5.9 |
| Vancouver | 6.2 | 0.1 | 12.3 | 0.4 | 1.2 | 0.2 | 24.5 | 0.8 |
| Victoria | 11.2 | 0.6 | 14.4 | 1.5 | 22.4 | 1.2 | 28.9 | 3.1 |

*R_0_ = basic reproductive rate; HR = hospitalization rate (as a percentage of symptomatic cases)

**Appendix D. Peak acute-care hospital demand, as percentage of capacity, by CMA and disease scenario.**

Results presented reflect the average value across five simulations. Confidence intervals were not included due to a very small degree of variance across simulations, which suggests that the timing of an outbreak — as it relates to school terms — has only a small impact on overall pandemic burden.

| **Census Metropolitan Area** | **Scenario** | | | | | | | | | | | |
| --- | --- | --- | --- | --- | --- | --- | --- | --- | --- | --- | --- | --- |
|  | **Ro=1.65; HR=0.4%*** | | | **Ro=1.80; HR=0.4%%** | | | **Ro=1.65; HR=1.0%** | | | **Ro=1.80; HR=1.0%** | | |
|  | **No Vaccination** | **25% Pre-vaccination** | **Reduction (%)** | **No Vaccination** | **25% Pre-vaccination** | **Reduction (%)** | **No Vaccination** | **25% Pre-vaccination** | **Reduction (%)** | **No Vaccination** | **25% Pre-vaccination** | **Reduction (%)** |
| St. John's | 3.6 | 0.4 | 88.9% | 4.6 | 0.8 | 82.6% | 7.3 | 0.8 | 89.0% | 9.2 | 1.5 | 83.7% |
| Halifax | 4.8 | 0.3 | 93.8% | 6.1 | 0.9 | 85.2% | 9.7 | 0.7 | 92.8% | 12.2 | 1.8 | 85.2% |
| Moncton | 3.6 | 0.4 | 88.9% | 4.5 | 0.8 | 82.2% | 7.2 | 0.9 | 87.5% | 9.1 | 1.5 | 83.5% |
| Saint John | 4.3 | 0.5 | 88.4% | 5.4 | 0.9 | 83.3% | 8.5 | 1.1 | 87.1% | 10.7 | 1.8 | 83.2% |
| Saguenay | 3.8 | 0.4 | 89.5% | 4.8 | 0.8 | 83.3% | 7.7 | 0.9 | 88.3% | 9.7 | 1.6 | 83.5% |
| Sherbrooke | 3.6 | 0.4 | 88.9% | 4.5 | 0.7 | 84.4% | 7.1 | 0.7 | 90.1% | 8.9 | 1.5 | 83.1% |
| Trois-Rivieres | 3.8 | 0.5 | 86.8% | 4.8 | 0.8 | 83.3% | 7.6 | 0.9 | 88.2% | 9.6 | 1.6 | 83.3% |
| Montreal | 4.6 | 0.1 | 97.8% | 7.9 | 0.2 | 97.5% | 9 | 0.1 | 98.9% | 15.8 | 0.5 | 96.8% |
| Quebec City | 4.9 | 0.2 | 95.9% | 6.1 | 0.7 | 88.5% | 9.7 | 0.4 | 95.9% | 12.3 | 1.3 | 89.4% |
| Ottawa-Gatineau | 7.9 | 0.2 | 97.5% | 10 | 0.8 | 92.0% | 15.8 | 0.4 | 97.5% | 20 | 1.5 | 92.5% |
| Kingston | 6.8 | 0.8 | 88.2% | 8.5 | 1.4 | 83.5% | 13.6 | 1.6 | 88.2% | 17.1 | 2.8 | 83.6% |
| Peterborough | 5.8 | 0.8 | 86.2% | 7.3 | 1.2 | 83.6% | 11.7 | 1.5 | 87.2% | 14.7 | 2.5 | 83.0% |
| Oshawa | 15.2 | 1.1 | 92.8% | 19.1 | 2.8 | 85.3% | 30.3 | 2.2 | 92.7% | 38.2 | 5.7 | 85.1% |
| Toronto | 7.8 | 0.1 | 98.7% | 16.4 | 0.4 | 97.6% | 15.1 | 0.2 | 98.7% | 32.7 | 0.7 | 97.9% |
| Hamilton | 8.4 | 0.4 | 95.2% | 10.5 | 1.2 | 88.6% | 16.7 | 0.7 | 95.8% | 21.1 | 2.3 | 89.1% |
| St. Catherine’s-Niagara | 12.8 | 0.9 | 93.0% | 16.1 | 2.3 | 85.7% | 25.5 | 1.8 | 92.9% | 32.1 | 4.6 | 85.7% |
| Windsor | 11.5 | 0.9 | 92.2% | 14.5 | 2.2 | 84.8% | 23 | 1.8 | 92.2% | 29 | 4.5 | 84.5% |
| Kitchener-Cambridge-Waterloo | 14.8 | 0.9 | 93.9% | 18.6 | 2.5 | 86.6% | 29.5 | 1.8 | 93.9% | 37.1 | 5 | 86.5% |
| Brantford | 15.3 | 1.9 | 87.6% | 19.1 | 3.2 | 83.2% | 60.5 | 3.8 | 93.7% | 38.4 | 6.4 | 83.3% |
| Guelph | 13.1 | 1.6 | 87.8% | 16.4 | 2.7 | 83.5% | 26.1 | 3.2 | 87.7% | 32.8 | 5.5 | 83.2% |
| London | 7 | 0.4 | 94.3% | 8.9 | 1.2 | 86.5% | 14.1 | 0.8 | 94.3% | 17.7 | 2.4 | 86.4% |
| Barrie | 12.2 | 1.3 | 89.3% | 15.3 | 2.5 | 83.7% | 24.2 | 2.6 | 89.3% | 30.5 | 5.1 | 83.3% |
| Greater Sudbury | 6.2 | 0.7 | 88.7% | 7.8 | 1.3 | 83.3% | 12.5 | 1.4 | 88.8% | 15.7 | 2.6 | 83.4% |
| Thunder Bay | 3.9 | 0.5 | 87.2% | 4.9 | 0.8 | 83.7% | 7.8 | 1 | 87.2% | 9.8 | 1.6 | 83.7% |
| Winnipeg | 5.6 | 0.2 | 96.4% | 7 | 0.8 | 88.6% | 11.2 | 0.5 | 95.5% | 14.1 | 1.5 | 89.4% |
| Regina | 5.1 | 0.5 | 90.2% | 6.4 | 1.1 | 82.8% | 10.2 | 1 | 90.2% | 12.9 | 2.1 | 83.7% |
| Saskatoon | 5.1 | 0.5 | 90.2% | 6.4 | 1 | 84.4% | 10.2 | 0.9 | 91.2% | 12.8 | 2.1 | 83.6% |
| Calgary | 7.7 | 0.2 | 97.4% | 9.7 | 0.8 | 91.8% | 15.4 | 0.4 | 97.4% | 19.5 | 1.5 | 92.3% |
| Edmonton | 6.8 | 0.2 | 97.1% | 8.6 | 0.7 | 91.9% | 13.6 | 0.4 | 97.1% | 17.2 | 1.4 | 91.9% |
| Kelowna | 7.9 | 0.9 | 88.6% | 9.9 | 1.6 | 83.8% | 15.8 | 1.7 | 89.2% | 19.8 | 3.3 | 83.3% |
| Abbotsford-Mission | 10.1 | 1.1 | 89.1% | 12.7 | 2.1 | 83.5% | 20.2 | 2.3 | 88.6% | 25.4 | 4.2 | 83.5% |
| Vancouver | 8.3 | 0.1 | 98.8% | 11.6 | 0.6 | 94.8% | 16.4 | 0.3 | 98.2% | 23.3 | 1.1 | 95.3% |
| Victoria | 5.6 | 0.4 | 92.9% | 7.1 | 1.1 | 84.5% | 11.3 | 0.8 | 92.9% | 14.2 | 2.1 | 85.2% |

*R_0_ = basic reproductive rate; HR = hospitalization rate (as a percentage of symptomatic cases)

**Appendix E. Cases of ICU admission, by CMA and disease scenario.**

Results presented reflect the average value across five simulations. Confidence intervals were not included due to a very small degree of variance across simulations, which suggests that the timing of an outbreak — as it relates to school terms — has only a small impact on overall pandemic burden. . Table E.1 presents percent reductions as a result of inter-wave vaccination, by CMA and disease scenario. Table E.2 presents ICU admission proportions, by CMA and disease scenario.

**Table E.1** Predicted number of ICU admissions by CMA, disease scenario and vaccination status.

| **Census Metropolitan Area** | **Scenario** | | | | | | | | | | | |
| --- | --- | --- | --- | --- | --- | --- | --- | --- | --- | --- | --- | --- |
|  | **Ro=1.65; HR=0.4%*** | | | **Ro=1.80; HR=0.4%%** | | | **Ro=1.65; HR=1.0%** | | | **Ro=1.80; HR=1.0%** | | |
|  | **No Vaccination** | **25% Pre-vaccination** | **Reduction (%)** | **No Vaccination** | **25% Pre-vaccination** | **Reduction (%)** | **No Vaccination** | **25% Pre-vaccination** | **Reduction (%)** | **No Vaccination** | **25% Pre-vaccination** | **Reduction (%)** |
| St. John's | 3.6 | 0.4 | 88.9% | 4.6 | 0.8 | 82.6% | 7.3 | 0.8 | 89.0% | 9.2 | 1.5 | 83.7% |
| Halifax | 4.8 | 0.3 | 93.8% | 6.1 | 0.9 | 85.2% | 9.7 | 0.7 | 92.8% | 12.2 | 1.8 | 85.2% |
| Moncton | 3.6 | 0.4 | 88.9% | 4.5 | 0.8 | 82.2% | 7.2 | 0.9 | 87.5% | 9.1 | 1.5 | 83.5% |
| Saint John | 4.3 | 0.5 | 88.4% | 5.4 | 0.9 | 83.3% | 8.5 | 1.1 | 87.1% | 10.7 | 1.8 | 83.2% |
| Saguenay | 3.8 | 0.4 | 89.5% | 4.8 | 0.8 | 83.3% | 7.7 | 0.9 | 88.3% | 9.7 | 1.6 | 83.5% |
| Sherbrooke | 3.6 | 0.4 | 88.9% | 4.5 | 0.7 | 84.4% | 7.1 | 0.7 | 90.1% | 8.9 | 1.5 | 83.1% |
| Trois-Rivieres | 3.8 | 0.5 | 86.8% | 4.8 | 0.8 | 83.3% | 7.6 | 0.9 | 88.2% | 9.6 | 1.6 | 83.3% |
| Montreal | 4.6 | 0.1 | 97.8% | 7.9 | 0.2 | 97.5% | 9 | 0.1 | 98.9% | 15.8 | 0.5 | 96.8% |
| Quebec City | 4.9 | 0.2 | 95.9% | 6.1 | 0.7 | 88.5% | 9.7 | 0.4 | 95.9% | 12.3 | 1.3 | 89.4% |
| Ottawa-Gatineau | 7.9 | 0.2 | 97.5% | 10 | 0.8 | 92.0% | 15.8 | 0.4 | 97.5% | 20 | 1.5 | 92.5% |
| Kingston | 6.8 | 0.8 | 88.2% | 8.5 | 1.4 | 83.5% | 13.6 | 1.6 | 88.2% | 17.1 | 2.8 | 83.6% |
| Peterborough | 5.8 | 0.8 | 86.2% | 7.3 | 1.2 | 83.6% | 11.7 | 1.5 | 87.2% | 14.7 | 2.5 | 83.0% |
| Oshawa | 15.2 | 1.1 | 92.8% | 19.1 | 2.8 | 85.3% | 30.3 | 2.2 | 92.7% | 38.2 | 5.7 | 85.1% |
| Toronto | 7.8 | 0.1 | 98.7% | 16.4 | 0.4 | 97.6% | 15.1 | 0.2 | 98.7% | 32.7 | 0.7 | 97.9% |
| Hamilton | 8.4 | 0.4 | 95.2% | 10.5 | 1.2 | 88.6% | 16.7 | 0.7 | 95.8% | 21.1 | 2.3 | 89.1% |
| St. Catharine’s-Niagara | 12.8 | 0.9 | 93.0% | 16.1 | 2.3 | 85.7% | 25.5 | 1.8 | 92.9% | 32.1 | 4.6 | 85.7% |
| Windsor | 11.5 | 0.9 | 92.2% | 14.5 | 2.2 | 84.8% | 23 | 1.8 | 92.2% | 29 | 4.5 | 84.5% |
| Kitchener-Cambridge-Waterloo | 14.8 | 0.9 | 93.9% | 18.6 | 2.5 | 86.6% | 29.5 | 1.8 | 93.9% | 37.1 | 5 | 86.5% |
| Brantford | 15.3 | 1.9 | 87.6% | 19.1 | 3.2 | 83.2% | 60.5 | 3.8 | 93.7% | 38.4 | 6.4 | 83.3% |
| Guelph | 13.1 | 1.6 | 87.8% | 16.4 | 2.7 | 83.5% | 26.1 | 3.2 | 87.7% | 32.8 | 5.5 | 83.2% |
| London | 7.0 | 0.4 | 94.3% | 8.9 | 1.2 | 86.5% | 14.1 | 0.8 | 94.3% | 17.7 | 2.4 | 86.4% |
| Barrie | 12.2 | 1.3 | 89.3% | 15.3 | 2.5 | 83.7% | 24.2 | 2.6 | 89.3% | 30.5 | 5.1 | 83.3% |
| Greater Sudbury | 6.2 | 0.7 | 88.7% | 7.8 | 1.3 | 83.3% | 12.5 | 1.4 | 88.8% | 15.7 | 2.6 | 83.4% |
| Thunder Bay | 3.9 | 0.5 | 87.2% | 4.9 | 0.8 | 83.7% | 7.8 | 1 | 87.2% | 9.8 | 1.6 | 83.7% |
| Winnipeg | 5.6 | 0.2 | 96.4% | 7 | 0.8 | 88.6% | 11.2 | 0.5 | 95.5% | 14.1 | 1.5 | 89.4% |
| Regina | 5.1 | 0.5 | 90.2% | 6.4 | 1.1 | 82.8% | 10.2 | 1 | 90.2% | 12.9 | 2.1 | 83.7% |
| Saskatoon | 5.1 | 0.5 | 90.2% | 6.4 | 1 | 84.4% | 10.2 | 0.9 | 91.2% | 12.8 | 2.1 | 83.6% |
| Calgary | 7.7 | 0.2 | 97.4% | 9.7 | 0.8 | 91.8% | 15.4 | 0.4 | 97.4% | 19.5 | 1.5 | 92.3% |
| Edmonton | 6.8 | 0.2 | 97.1% | 8.6 | 0.7 | 91.9% | 13.6 | 0.4 | 97.1% | 17.2 | 1.4 | 91.9% |
| Kelowna | 7.9 | 0.9 | 88.6% | 9.9 | 1.6 | 83.8% | 15.8 | 1.7 | 89.2% | 19.8 | 3.3 | 83.3% |
| Abbotsford-Mission | 10.1 | 1.1 | 89.1% | 12.7 | 2.1 | 83.5% | 20.2 | 2.3 | 88.6% | 25.4 | 4.2 | 83.5% |
| Vancouver | 8.3 | 0.1 | 98.8% | 11.6 | 0.6 | 94.8% | 16.4 | 0.3 | 98.2% | 23.3 | 1.1 | 95.3% |
| Victoria | 5.6 | 0.4 | 92.9% | 7.1 | 1.1 | 84.5% | 11.3 | 0.8 | 92.9% | 14.2 | 2.1 | 85.2% |

*R_0_ = basic reproductive rate; HR = hospitalization rate (as a percentage of symptomatic cases)

**Table E.2** Predicted proportion of ICU admissions (per 10,000 population) by CMA, disease scenario and vaccination status.

| **Census Metropolitan Area** | **Scenario** | | | | | | | |
| --- | --- | --- | --- | --- | --- | --- | --- | --- |
|  | **Ro=1.65; HR=0.4%** | | **Ro=1.80; HR=0.4%%** | | **Ro=1.65; HR=1.0%** | | **Ro=1.80; HR=1.0%** | |
|  | **No Vaccination** | **25% Pre-vaccination** | **No Vaccination** | **25% Pre-vaccination** | **No Vaccination** | **25% Pre-vaccination** | **No Vaccination** | **25% Pre-vaccination** |
| St. John's | 3.1 | 0.3 | 3.8 | 0.5 | 6.2 | 0.5 | 7.5 | 1.1 |
| Halifax | 2.8 | 0.1 | 3.7 | 0.4 | 5.7 | 0.3 | 7.5 | 0.7 |
| Moncton | 3.5 | 0.4 | 4.2 | 0.6 | 7.1 | 0.6 | 8.4 | 1.4 |
| Saint John | 3.8 | 0.4 | 4.3 | 0.7 | 7.4 | 0.7 | 8.7 | 1.4 |
| Saguenay | 3.4 | 0.3 | 3.9 | 0.6 | 6.7 | 0.6 | 7.9 | 1.2 |
| Sherbrooke | 3.3 | 0.2 | 4.0 | 0.5 | 6.6 | 0.5 | 8.0 | 1.1 |
| Trois-Rivieres | 3.4 | 0.3 | 4.0 | 0.6 | 6.7 | 0.6 | 7.9 | 1.3 |
| Montreal | 1.0 | 0.0 | 2.5 | 0.1 | 2.0 | 0.0 | 4.9 | 0.1 |
| Quebec City | 2.5 | 0.1 | 3.6 | 0.2 | 4.9 | 0.2 | 7.3 | 0.5 |
| Ottawa-Gatineau | 2.0 | 0.0 | 3.4 | 0.2 | 4.0 | 0.1 | 6.8 | 0.3 |
| Kingston | 3.4 | 0.3 | 4.1 | 0.6 | 6.9 | 0.6 | 8.1 | 1.3 |
| Peterborough | 3.6 | 0.3 | 4.2 | 0.8 | 7.3 | 0.8 | 8.5 | 1.4 |
| Oshawa | 3.4 | 0.2 | 4.4 | 0.4 | 6.7 | 0.3 | 8.7 | 0.9 |
| Toronto | 0.8 | 0.0 | 2.2 | 0.0 | 1.6 | 0.0 | 4.4 | 0.1 |
| Hamilton | 2.7 | 0.1 | 4.0 | 0.3 | 5.3 | 0.2 | 7.9 | 0.5 |
| St. Catharines-Niagara | 3.3 | 0.2 | 4.3 | 0.4 | 6.5 | 0.3 | 8.5 | 0.8 |
| Windsor | 3.7 | 0.2 | 4.7 | 0.5 | 7.4 | 0.4 | 9.4 | 1.0 |
| Kitchener-Cambridge-Waterloo | 2.9 | 0.1 | 3.9 | 0.3 | 5.7 | 0.3 | 7.8 | 0.7 |
| Brantford | 3.8 | 0.4 | 4.4 | 0.7 | 7.5 | 0.7 | 8.9 | 1.4 |
| Guelph | 3.5 | 0.4 | 4.2 | 0.6 | 7.1 | 0.6 | 8.4 | 1.3 |
| London | 2.9 | 0.1 | 4.0 | 0.3 | 5.8 | 0.3 | 7.9 | 0.7 |
| Barrie | 3.8 | 0.3 | 4.6 | 0.6 | 7.6 | 0.6 | 9.3 | 1.3 |
| Greater Sudbury | 3.5 | 0.3 | 4.2 | 0.6 | 7.2 | 0.6 | 8.5 | 1.3 |
| Thunder Bay | 3.7 | 0.3 | 4.3 | 0.7 | 7.4 | 0.7 | 8.6 | 1.5 |
| Winnipeg | 2.6 | 0.1 | 3.8 | 0.3 | 5.2 | 0.2 | 7.7 | 0.5 |
| Regina | 3.2 | 0.2 | 3.8 | 0.5 | 6.4 | 0.5 | 7.7 | 1.0 |
| Saskatoon | 3.0 | 0.2 | 3.8 | 0.5 | 6.1 | 0.4 | 7.5 | 0.9 |
| Calgary | 1.9 | 0.0 | 3.2 | 0.2 | 3.8 | 0.1 | 6.3 | 0.3 |
| Edmonton | 2.0 | 0.1 | 3.2 | 0.2 | 3.9 | 0.1 | 6.4 | 0.3 |
| Kelowna | 3.5 | 0.3 | 4.2 | 0.6 | 7.1 | 0.6 | 8.6 | 1.2 |
| Abbotsford-Mission | 3.6 | 0.3 | 4.3 | 0.6 | 7.3 | 0.6 | 8.7 | 1.3 |
| Vancouver | 1.5 | 0.0 | 3.0 | 0.1 | 2.8 | 0.1 | 5.9 | 0.2 |
| Victoria | 2.8 | 0.1 | 3.6 | 0.4 | 5.5 | 0.3 | 7.2 | 0.7 |

**Appendix F. Peak ICU demand, as percentage of capacity, by CMA and disease scenario.**

Results presented reflect the average value across five simulations. Confidence intervals were not included due to a very small degree of variance across simulations, which suggests that the timing of an outbreak — as it relates to school terms — has only a small impact on overall pandemic burden.

| **Census Metropolitan Area** | **Scenario** | | | | | | | | | | | |
| --- | --- | --- | --- | --- | --- | --- | --- | --- | --- | --- | --- | --- |
|  | **Ro=1.65; HR=0.4%*** | | | **Ro=1.80; HR=0.4%%** | | | **Ro=1.65; HR=1.0%** | | | **Ro=1.80; HR=1.0%** | | |
|  | **No Vaccination** | **25% Pre-vaccination** | **Reduction (%)** | **No Vaccination** | **25% Pre-vaccination** | **Reduction (%)** | **No Vaccination** | **25% Pre-vaccination** | **Reduction (%)** | **No Vaccination** | **25% Pre-vaccination** | **Reduction (%)** |
| St. John's | 15.1 | 1.4 | 90.7% | 18.8 | 3.1 | 83.5% | 30.3 | 2.9 | 90.4% | 37.7 | 6.2 | 83.6% |
| Halifax | 26.2 | 1.6 | 93.9% | 32.5 | 4.3 | 86.8% | 52.5 | 3.1 | 94.1% | 65.3 | 8.6 | 86.8% |
| Moncton | 25.1 | 2.9 | 88.4% | 31.1 | 5.3 | 83.0% | 50.2 | 5.7 | 88.6% | 62.4 | 10.7 | 82.9% |
| Saint John | 22.8 | 2.7 | 88.2% | 28.3 | 4.8 | 83.0% | 45.5 | 5.4 | 88.1% | 56.6 | 9.7 | 82.9% |
| Saguenay | 14.1 | 1.5 | 89.4% | 17.5 | 3.0 | 82.9% | 28.2 | 3.0 | 89.4% | 35.1 | 6.0 | 82.9% |
| Sherbrooke | 12.3 | 1.2 | 90.2% | 15.3 | 2.5 | 83.7% | 24.6 | 2.3 | 90.7% | 30.6 | 5.0 | 83.7% |
| Trois-Rivieres | 11.0 | 1.2 | 89.1% | 13.6 | 2.3 | 83.1% | 22.0 | 2.4 | 89.1% | 27.3 | 4.7 | 82.8% |
| Montreal | 9.7 | 0.1 | 99.0% | 19.9 | 0.5 | 97.5% | 18.9 | 0.3 | 98.4% | 39.7 | 1.0 | 97.5% |
| Quebec City | 13.0 | 0.5 | 96.2% | 16.2 | 1.5 | 90.7% | 26.0 | 0.9 | 96.5% | 32.4 | 2.9 | 91.0% |
| Ottawa-Gatineau | 49.8 | 1.2 | 97.6% | 65.6 | 4.2 | 93.6% | 98.6 | 2.4 | 97.6% | 131.3 | 8.3 | 93.7% |
| Kingston | 16.1 | 1.7 | 89.4% | 20.0 | 3.4 | 83.0% | 32.2 | 3.4 | 89.4% | 40.1 | 6.8 | 83.0% |
| Peterborough | 35.2 | 4.3 | 87.8% | 43.7 | 7.5 | 82.8% | 70.5 | 8.6 | 87.8% | 87.6 | 15.1 | 82.8% |
| Oshawa | 82.7 | 5.3 | 93.6% | 102.7 | 14.1 | 86.3% | 164.9 | 10.5 | 93.6% | 205.3 | 28.2 | 86.3% |
| Toronto | 28.7 | 0.3 | 99.0% | 70.4 | 1.3 | 98.2% | 55.5 | 0.7 | 98.7% | 139.3 | 2.6 | 98.1% |
| Hamilton | 33.2 | 1.2 | 96.4% | 41.2 | 3.9 | 90.5% | 66.3 | 11.6 | 82.5% | 82.6 | 7.7 | 90.7% |
| St. Catherine’s-Niagara | 97.9 | 5.8 | 94.1% | 121.5 | 16.0 | 86.8% | 195.7 | 7.5 | 96.2% | 243.4 | 32.0 | 86.9% |
| Windsor | 54.6 | 3.8 | 93.0% | 67.8 | 9.7 | 85.7% | 108.8 | 7.5 | 93.1% | 135.5 | 19.4 | 85.7% |
| Kitchener-Cambridge-Waterloo | 68.4 | 3.5 | 94.9% | 84.9 | 10.2 | 88.0% | 136.6 | 7.0 | 94.9% | 170.2 | 20.3 | 88.1% |
| Brantford | 67.8 | 7.8 | 88.5% | 84.2 | 14.4 | 82.9% | 135.4 | 15.6 | 88.5% | 168.5 | 28.9 | 82.8% |
| Guelph | 45.2 | 5.1 | 88.7% | 56.1 | 9.5 | 83.1% | 90.2 | 10.2 | 88.7% | 112.3 | 19.2 | 82.9% |
| London | 28.7 | 1.5 | 94.8% | 35.7 | 4.3 | 88.0% | 57.4 | 2.9 | 94.9% | 71.5 | 8.6 | 88.0% |
| Barrie | 70.1 | 6.8 | 90.3% | 87.0 | 14.4 | 83.4% | 139.7 | 13.6 | 90.3% | 174.0 | 28.9 | 83.4% |
| Greater Sudbury | 50.0 | 5.3 | 89.4% | 62.1 | 10.5 | 83.1% | 100.1 | 10.6 | 89.4% | 124.4 | 21.1 | 83.0% |
| Thunder Bay | 31.3 | 3.8 | 87.9% | 38.7 | 6.6 | 82.9% | 62.5 | 7.8 | 87.5% | 77.7 | 13.4 | 82.8% |
| Winnipeg | 23.0 | 0.8 | 96.5% | 28.5 | 2.7 | 90.5% | 45.8 | 1.6 | 96.5% | 57.1 | 5.3 | 90.7% |
| Regina | 35.1 | 3.2 | 90.9% | 43.5 | 7.0 | 83.9% | 70.1 | 6.4 | 90.9% | 87.2 | 14.1 | 83.8% |
| Saskatoon | 37.8 | 3.0 | 92.1% | 47.0 | 7.2 | 84.7% | 75.6 | 6.0 | 92.1% | 94.2 | 14.4 | 84.7% |
| Calgary | 28.2 | 0.7 | 97.5% | 36.9 | 2.4 | 93.5% | 55.9 | 1.4 | 97.5% | 74.0 | 4.8 | 93.5% |
| Edmonton | 26.9 | 0.7 | 97.4% | 34.9 | 2.4 | 93.1% | 53.4 | 1.3 | 97.6% | 70.0 | 4.7 | 93.3% |
| Kelowna | 41.7 | 4.2 | 89.9% | 51.8 | 8.6 | 83.4% | 85.5 | 8.3 | 90.3% | 103.8 | 17.3 | 83.3% |
| Abbotsford-Mission | 79.1 | 8.1 | 89.8% | 98.3 | 16.4 | 83.3% | 157.8 | 16.2 | 89.7% | 196.6 | 33.0 | 83.2% |
| Vancouver | 52.7 | 0.9 | 98.3% | 85.2 | 3.3 | 96.1% | 103.6 | 1.7 | 98.4% | 170.9 | 6.5 | 96.2% |
| Victoria | 62.0 | 4.0 | 93.5% | 76.9 | 10.6 | 86.2% | 124.3 | 8.0 | 93.6% | 154.5 | 21.2 | 86.3% |

*R_0_ = basic reproductive rate; HR = hospitalization rate (as a percentage of symptomatic cases)

**Appendix G. Total influenza-related mortality, by CMA and disease scenario.**

Results presented reflect the average value across five simulations. Confidence intervals were not included due to a very small degree of variance across simulations, which suggests that the timing of an outbreak — as it relates to school terms — has only a small impact on overall pandemic burden.

| **Census Metropolitan Area** | **Scenario** | | | | | | | | | | | |
| --- | --- | --- | --- | --- | --- | --- | --- | --- | --- | --- | --- | --- |
|  | **Ro=1.65; HR=0.4%*** | | | **Ro=1.80; HR=0.4%%** | | | **Ro=1.65; HR=1.0%** | | | **Ro=1.80; HR=1.0%** | | |
|  | **No Vaccination** | **25% Pre-vaccination** | **Reduction (%)** | **No Vaccination** | **25% Pre-vaccination** | **Reduction (%)** | **No Vaccination** | **25% Pre-vaccination** | **Reduction (%)** | **No Vaccination** | **25% Pre-vaccination** | **Reduction (%)** |
| St. John's | 36 | 2 | 94.4% | 46 | 5 | 89.1% | 71 | 5 | 93.0% | 92 | 11 | 88.0% |
| Halifax | 57 | 3 | 94.7% | 82 | 7 | 91.5% | 113 | 5 | 95.6% | 164 | 13 | 92.1% |
| Moncton | 30 | 2 | 93.3% | 37 | 5 | 86.5% | 60 | 5 | 91.7% | 74 | 10 | 86.5% |
| Saint John | 29 | 2 | 93.1% | 36 | 5 | 86.1% | 57 | 5 | 91.2% | 71 | 10 | 85.9% |
| Saguenay | 30 | 2 | 93.3% | 38 | 5 | 86.8% | 60 | 5 | 91.7% | 77 | 10 | 87.0% |
| Sherbrooke | 41 | 3 | 92.7% | 53 | 6 | 88.7% | 81 | 5 | 93.8% | 105 | 12 | 88.6% |
| Trois-Rivieres | 29 | 2 | 93.1% | 36 | 5 | 86.1% | 58 | 4 | 93.1% | 73 | 10 | 86.3% |
| Montreal | 185 | 3 | 98.4% | 483 | 11 | 97.7% | 359 | 7 | 98.1% | 951 | 21 | 97.8% |
| Quebec City | 98 | 3 | 96.9% | 163 | 9 | 94.5% | 193 | 6 | 96.9% | 325 | 18 | 94.5% |
| Ottawa-Gatineau | 126 | 3 | 97.6% | 238 | 10 | 95.8% | 246 | 6 | 97.6% | 472 | 19 | 96.0% |
| Kingston | 31 | 2 | 93.5% | 39 | 5 | 87.2% | 62 | 5 | 91.9% | 79 | 10 | 87.3% |
| Peterborough | 26 | 2 | 92.3% | 31 | 5 | 83.9% | 51 | 4 | 92.2% | 63 | 9 | 85.7% |
| Oshawa | 71 | 3 | 95.8% | 100 | 9 | 91.0% | 139 | 7 | 95.0% | 199 | 17 | 91.5% |
| Toronto | 199 | 3 | 98.5% | 578 | 11 | 98.1% | 385 | 7 | 98.2% | 1130 | 22 | 98.1% |
| Hamilton | 101 | 3 | 97.0% | 167 | 9 | 94.6% | 200 | 7 | 96.5% | 332 | 19 | 94.3% |
| St. Catherine’s-Niagara | 66 | 3 | 95.5% | 95 | 8 | 91.6% | 130 | 6 | 95.4% | 190 | 15 | 92.1% |
| Windsor | 67 | 3 | 95.5% | 94 | 9 | 90.4% | 113 | 7 | 93.8% | 187 | 17 | 90.9% |
| Kitchener-Cambridge-Waterloo | 81 | 3 | 96.3% | 120 | 9 | 92.5% | 159 | 6 | 96.2% | 240 | 17 | 92.9% |
| Brantford | 34 | 3 | 91.2% | 42 | 6 | 85.7% | 68 | 6 | 91.2% | 85 | 12 | 85.9% |
| Guelph | 32 | 3 | 90.6% | 40 | 5 | 87.5% | 64 | 5 | 92.2% | 80 | 11 | 86.3% |
| London | 77 | 3 | 96.1% | 116 | 8 | 93.1% | 153 | 6 | 96.1% | 231 | 17 | 92.6% |
| Barrie | 44 | 3 | 93.2% | 57 | 7 | 87.7% | 87 | 6 | 93.1% | 113 | 14 | 87.6% |
| Greater Sudbury | 33 | 2 | 93.9% | 42 | 5 | 88.1% | 66 | 5 | 92.4% | 84 | 11 | 86.9% |
| Thunder Bay | 25 | 2 | 92.0% | 31 | 4 | 87.1% | 50 | 4 | 92.0% | 62 | 9 | 85.5% |
| Winnipeg | 100 | 3 | 97.0% | 166 | 9 | 94.6% | 198 | 6 | 97.0% | 330 | 18 | 94.5% |
| Regina | 45 | 3 | 93.3% | 58 | 7 | 87.9% | 89 | 6 | 93.3% | 117 | 13 | 88.9% |
| Saskatoon | 52 | 3 | 94.2% | 69 | 7 | 89.9% | 102 | 6 | 94.1% | 139 | 14 | 89.9% |
| Calgary | 127 | 3 | 97.6% | 238 | 10 | 95.8% | 249 | 7 | 97.2% | 472 | 20 | 95.8% |
| Edmonton | 124 | 3 | 97.6% | 229 | 10 | 95.6% | 243 | 6 | 97.5% | 455 | 19 | 95.8% |
| Kelowna | 35 | 2 | 94.3% | 45 | 5 | 88.9% | 70 | 5 | 92.9% | 90 | 11 | 87.8% |
| Abbotsford-Mission | 43 | 3 | 93.0% | 54 | 7 | 87.0% | 85 | 6 | 92.9% | 109 | 14 | 87.2% |
| Vancouver | 139 | 3 | 97.8% | 315 | 9 | 97.1% | 272 | 6 | 97.8% | 624 | 18 | 97.1% |
| Victoria | 45 | 2 | 95.6% | 65 | 6 | 90.8% | 90 | 4 | 95.6% | 129 | 11 | 91.5% |

*R_0_ = basic reproductive rate; HR = hospitalization rate (as a percentage of symptomatic cases)

**Appendix H. Sensitivity analysis of demographic and health system predictors of elevated pandemic burden and hospital-resource stress.**

Scenario 1: R_0_ = 1.65, hospitalization rate = 0.4%; no intervention; pre-existing immunity in place; Scenario 2 R_0_ = 1.80, hospitalization rate = 0.4%; no intervention; pre-existing immunity in place; Scenario 3: R_0_ = 1.65, hospitalization rate = 1.0%; no intervention; pre-existing immunity in place; Scenario 4: R_0_ = 1.65, hospitalization rate = 0.4%; 25% pre-vaccination; pre-existing immunity in place; Scenario 5: R_0_ = 1.65, hospitalization rate = 0.4%; no intervention; no pre-existing immunity.

| **Characteristic** | **Scenario** | **Symptomatic cases** | | **Hospitalizations** | | **ICU cases** | | **Peak acute care demand (%)** | | **Peak ICU demand (%)** | | **Deaths** | |
| --- | --- | --- | --- | --- | --- | --- | --- | --- | --- | --- | --- | --- | --- |
|  |  | **Correlation (R)** | **r^2^** | **Correlation (R)** | **r^2^** | **Correlation (R)** | **r^2^** | **Correlation (R)** | **r^2^** | **Correlation (R)** | **r^2^** | **Correlation (R)** | **r^2^** |
| **% Infants** | 1 | 0.2486 | 0.0618 | 0.2763 | 0.0763 | 0.2535 | 0.0643 | 0.2944 | 0.0866 | 0.0802 | 0.0064 | 0.3558 | 0.1266 |
|  | 2 | 0.2103 | 0.0442 | 0.2265 | 0.0513 | 0.2117 | 0.0448 | 0.3083 | 0.9500 | 0.0933 | 0.0087 | 0.2788 | 0.0777 |
|  | 3 | 0.2495 | 0.0442 | 0.3607 | 0.0513 | 0.2540 | 0.0448 | 0.2920 | 0.0950 | 0.0756 | 0.0087 | 0.3552 | 0.0777 |
|  | 4 | 0.2646 | 0.0700 | 0.4001 | 0.1601 | 0.3723 | 0.1386 | 0.0784 | 0.0061 | 0.0103 | 0.0001 | 0.6598 | 0.4353 |
|  | 5 | 0.1358 | 0.0185 | 0.0577 | 0.0033 | 0.0527 | 0.0028 | 0.0903 | 0.0082 | 0.1120 | 0.0019 | 0.0640 | 0.0041 |
| **% Children** | 1 | 0.0537 | 0.0029 | 0.1143 | 0.1310 | 0.1022 | 0.0104 | 0.6761 | 0.4572 | 0.4955 | 0.2456 | 0.1718 | 0.0295 |
|  | 2 | 0.0519 | 0.0027 | 0.0835 | 0.0070 | 0.0752 | 0.0057 | 0.6757 | 0.4566 | 0.4995 | 0.2495 | 0.1100 | 0.0121 |
|  | 3 | 0.0531 | 0.0028 | 0.1702 | 0.0290 | 0.1023 | 0.0105 | 0.6735 | 0.3181 | 0.4915 | 0.2416 | 0.1581 | 0.0250 |
|  | 4 | 0.0632 | 0.0040 | 0.4733 | 0.2240 | 0.5511 | 0.3037 | 0.4610 | 0.2125 | 0.4249 | 0.1805 | 0.6122 | 0.3748 |
|  | 5 | 0.0000 | 0.0000 | -0.0636 | 0.0040 | -0.0584 | 0.0034 | 0.4061 | 0.1649 | 0.5129 | 0.0879 | -0.0712 | 0.0052 |
| **% Young adults** | 1 | 0.1087 | 0.0118 | 0.0776 | 0.0060 | 0.0791 | 0.0063 | -0.2222 | 0.0476 | -0.0752 | 0.0057 | 0.0877 | 0.0077 |
|  | 2 | 0.0658 | 0.0043 | 0.0499 | 0.0025 | 0.0508 | 0.0026 | -0.2160 | 0.0466 | -0.0683 | 0.0047 | 0.0625 | 0.0039 |
|  | 3 | 0.1109 | 0.0123 | 0.0639 | 0.0041 | 0.0806 | 0.0065 | -0.2194 | 0.0543 | -0.0752 | 0.0057 | 0.0992 | 0.0098 |
|  | 4 | 0.2297 | 0.0527 | -0.0617 | 0.0038 | -0.0837 | 0.0070 | -0.2788 | 0.0778 | -0.2043 | 0.0417 | 0.0473 | 0.0022 |
|  | 5 | 0.1362 | 0.0186 | 0.2476 | 0.0613 | 0.2351 | 0.0553 | 0.1360 | 0.0185 | -0.0646 | 0.0310 | 0.2754 | 0.0759 |
| **% Adults** | 1 | 0.3185 | 0.1014 | 0.3128 | 0.0979 | 0.3137 | 0.0984 | -0.1279 | 0.0164 | -0.3176 | 0.1009 | 0.3029 | 0.0918 |
|  | 2 | 0.2999 | 0.0895 | 0.2985 | 0.0891 | 0.3013 | 0.0908 | -0.0770 | 0.0059 | -0.2483 | 0.0617 | 0.3046 | 0.0928 |
|  | 3 | 0.3186 | 0.1015 | 0.2016 | 0.0406 | 0.3130 | 0.0980 | -0.1321 | 0.0169 | -0.3230 | 0.1043 | 0.3020 | 0.0912 |
|  | 4 | 0.2208 | 0.0487 | 0.1777 | 0.0316 | 0.1621 | 0.0263 | -0.3076 | 0.0946 | -0.4029 | 0.1623 | 0.0245 | 0.0006 |
|  | 5 | 0.1939 | 0.0376 | 0.0622 | 0.0039 | 0.0594 | 0.0035 | -0.3690 | 0.1362 | -0.2403 | 0.1793 | 0.0401 | 0.0016 |
| **% Seniors** | 1 | -0.3774 | 0.1424 | -0.3934 | 0.1547 | -0.3924 | 0.1462 | -0.2245 | 0.0503 | -0.0512 | 0.0027 | -0.4475 | 0.2002 |
|  | 2 | -0.3230 | 0.1044 | -0.3336 | 0.1130 | -0.3276 | 0.1073 | -0.2649 | 0.0702 | -0.1073 | 0.0115 | -0.3749 | 0.1406 |
|  | 3 | -0.3790 | 0.1436 | -0.3645 | 0.1328 | -0.3823 | 0.1469 | -0.2215 | 0.0201 | -0.0449 | 0.0020 | -0.4471 | 0.1999 |
|  | 4 | -0.4130 | 0.1705 | -0.4454 | 0.1984 | -0.4586 | 0.2103 | 0.1129 | 0.0127 | 0.1572 | 0.0247 | -0.5709 | 0.3260 |
|  | 5 | -0.2584 | 0.0668 | -0.1990 | 0.0396 | -0.1898 | 0.0360 | -0.1238 | 0.0153 | -0.1276 | 0.0004 | -0.2020 | 0.0408 |
| **Acute care beds (/10,000)** | 1 | NA | NA | NA | NA | NA | NA | -0.8697 | 0.7564 | -0.7044 | 0.4962 | NA | NA |
|  | 2 | NA | NA | NA | NA | NA | NA | -0.9077 | 0.8238 | -0.7629 | 0.5820 | NA | NA |
|  | 3 | NA | NA | NA | NA | NA | NA | -0.8676 | 0.7527 | -0.7012 | 0.4917 | NA | NA |
|  | 4 | NA | NA | NA | NA | NA | NA | -0.4628 | 0.2142 | -0.3876 | 0.1502 | NA | NA |
|  | 5 | NA | NA | NA | NA | NA | NA | -0.9127 | 0.8330 | -0.7709 | 0.4581 | NA | NA |
| **ICU beds (/10,000)** | 1 | NA | NA | NA | NA | NA | NA | -0.6592 | 0.4345 | -0.8151 | 0.6644 | NA | NA |
|  | 2 | NA | NA | NA | NA | NA | NA | -0.6720 | 0.4515 | -0.8502 | 0.7228 | NA | NA |
|  | 3 | NA | NA | NA | NA | NA | NA | -0.6599 | 0.2879 | -0.8133 | 0.6614 | NA | NA |
|  | 4 | NA | NA | NA | NA | NA | NA | -0.3861 | 0.1491 | -0.5742 | 0.3297 | NA | NA |
|  | 5 | NA | NA | NA | NA | NA | NA | -0.6969 | 0.4857 | -0.8529 | 0.6470 | NA | NA |

**Appendix I. Sensitivity analysis scatter plots**

This appendix presents five unique scatterplots, and associated r^2^ value, for each of the 34 associations between demographic and health system characteristics and pandemic outcomes tested.

**Figure I.1.** Association between the percentage of the total population represented by infants and the overall number of symptomatic cases. Negligible correlation in Scenario 1 (A), Scenario 2 (B), Scenario 3 (C), Scenario 4 (D) and Scenario 5 (E). Scenario 1: R_0_ = 1.65, hospitalization rate = 0.4%; no intervention; pre-existing immunity in place; Scenario 2 R_0_ = 1.80, hospitalization rate = 0.4%; no intervention; pre-existing immunity in place; Scenario 3: R_0_ = 1.65, hospitalization rate = 1.0%; no intervention; pre-existing immunity in place; Scenario 4: R_0_ = 1.65, hospitalization rate = 0.4%; 25% pre-vaccination; pre-existing immunity in place; Scenario 5: R_0_ = 1.65, hospitalization rate = 0.4%; no intervention; no pre-existing immunity.

**A)**

**B)**

**C)**

**D)**

**E)**

**Figure I.2.** Association between the percentage of the total population represented by infants and the overall number of acute hospitalizations admissions. Negligible correlation in Scenario 1 (A), Scenario 2 (B) and Scenario 5 (E); mild correlation in Scenario 3 (C) and 4 (D). Scenario 1: R_0_ = 1.65, hospitalization rate = 0.4%; no intervention; pre-existing immunity in place; Scenario 2 R_0_ = 1.80, hospitalization rate = 0.4%; no intervention; pre-existing immunity in place; Scenario 3: R_0_ = 1.65, hospitalization rate = 1.0%; no intervention; pre-existing immunity in place; Scenario 4: R_0_ = 1.65, hospitalization rate = 0.4%; 25% pre-vaccination; pre-existing immunity in place; Scenario 5: R_0_ = 1.65, hospitalization rate = 0.4%; no intervention; no pre-existing immunity.

**A)**

**B)**

**C)**

**D)**

**E)**

**Figure I.3.** Association between the percentage of the total population represented by infants and the overall number of ICU admissions. Negligible correlation in Scenario 1 (A), Scenario 2 (B), Scenario 3 (C) and Scenario 5 (E); mild correlation in Scenario 4 (D). Scenario 1: R_0_ = 1.65, hospitalization rate = 0.4%; no intervention; pre-existing immunity in place; Scenario 2 R_0_ = 1.80, hospitalization rate = 0.4%; no intervention; pre-existing immunity in place; Scenario 3: R_0_ = 1.65, hospitalization rate = 1.0%; no intervention; pre-existing immunity in place; Scenario 4: R_0_ = 1.65, hospitalization rate = 0.4%; 25% pre-vaccination; pre-existing immunity in place; Scenario 5: R_0_ = 1.65, hospitalization rate = 0.4%; no intervention; no pre-existing immunity.

**A)**

**B)**

**C)**

**D)**

**E)**

**Figure I.4.** Association between the percentage of the total population represented by infants and the peak acute-care demand (as a percentage of total capacity). Negligible correlation in Scenario 1 (A), Scenario 3 (C), Scenario 4 (D) and Scenario 5 (E); mild correlation in Scenario 2 (B). Scenario 1: R_0_ = 1.65, hospitalization rate = 0.4%; no intervention; pre-existing immunity in place; Scenario 2 R_0_ = 1.80, hospitalization rate = 0.4%; no intervention; pre-existing immunity in place; Scenario 3: R_0_ = 1.65, hospitalization rate = 1.0%; no intervention; pre-existing immunity in place; Scenario 4: R_0_ = 1.65, hospitalization rate = 0.4%; 25% pre-vaccination; pre-existing immunity in place; Scenario 5: R_0_ = 1.65, hospitalization rate = 0.4%; no intervention; no pre-existing immunity.

**A)**

**B)**

**
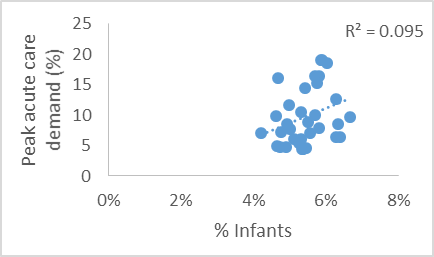
**

**C)**

**
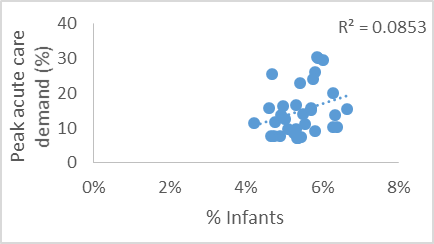
**

**D)**

**
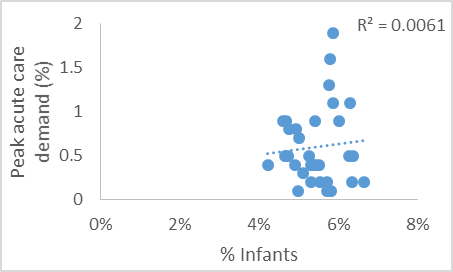
**

**E)**

**
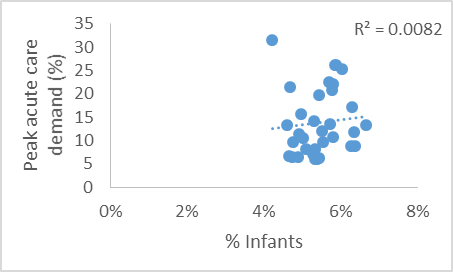
**

**Figure I.5.** Association between the percentage of the total population represented by infants and the peak ICU demand (as a percentage of total capacity). Negligible correlation in Scenario 1 (A), Scenario 2 (B), Scenario 3 (C), Scenario 4 (D) and Scenario 5 (E). Scenario 1: R_0_ = 1.65, hospitalization rate = 0.4%; no intervention; pre-existing immunity in place; Scenario 2 R_0_ = 1.80, hospitalization rate = 0.4%; no intervention; pre-existing immunity in place; Scenario 3: R_0_ = 1.65, hospitalization rate = 1.0%; no intervention; pre-existing immunity in place; Scenario 4: R_0_ = 1.65, hospitalization rate = 0.4%; 25% pre-vaccination; pre-existing immunity in place; Scenario 5: R_0_ = 1.65, hospitalization rate = 0.4%; no intervention; no pre-existing immunity.

**A)**

**
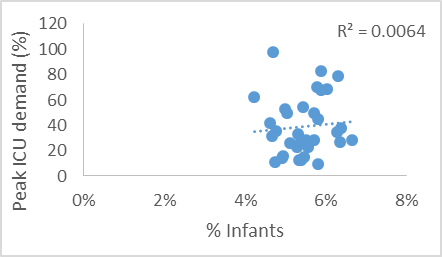
**

**B)**

**
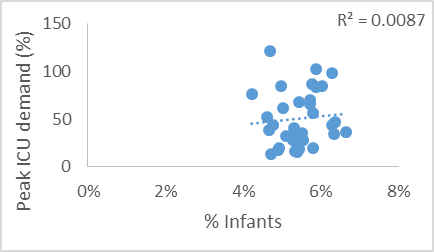
**

**C)**

**
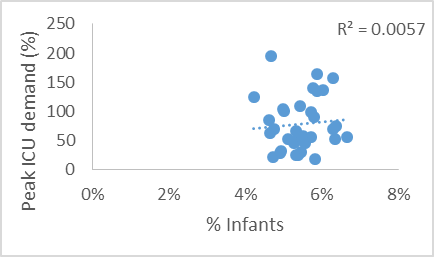
**

**D)**

**E)**


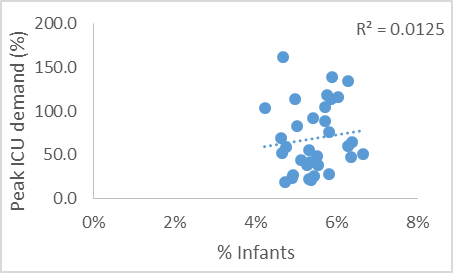


**Figure I.6.** Association between the percentage of the total population represented by infants and total mortality. Negligible correlation in Scenario 2 (B) and Scenario 5 (E); mild correlation in Scenario 1 (A) Scenario 3 (C); moderate correlation in Scenario 4 (D). Scenario 1: R_0_ = 1.65, hospitalization rate = 0.4%; no intervention; pre-existing immunity in place; Scenario 2 R_0_ = 1.80, hospitalization rate = 0.4%; no intervention; pre-existing immunity in place; Scenario 3: R_0_ = 1.65, hospitalization rate = 1.0%; no intervention; pre-existing immunity in place; Scenario 4: R_0_ = 1.65, hospitalization rate = 0.4%; 25% pre-vaccination; pre-existing immunity in place; Scenario 5: R_0_ = 1.65, hospitalization rate = 0.4%; no intervention; no pre-existing immunity.

**A)**

**
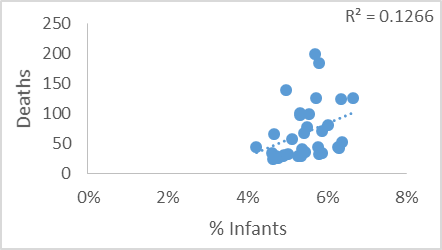
**

**B)**

**
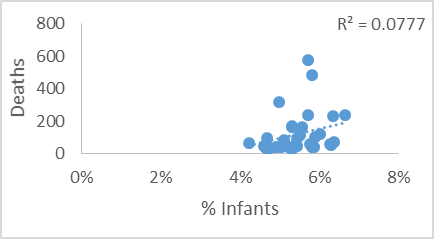
**

**C)**

**
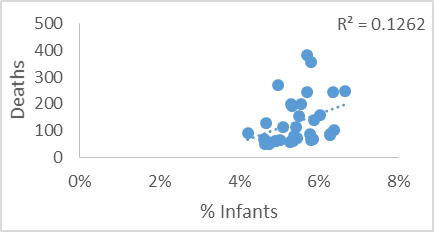
**

**D)**

**
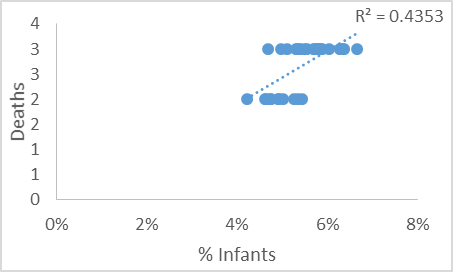
**

**E)**


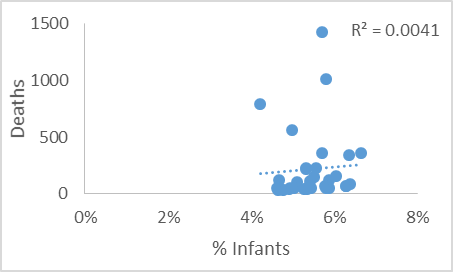


**Figure I.7.** Association between the percentage of the total population represented by children and overall number of symptomatic cases. Negligible correlation in Scenario 1 (A), Scenario 2 (B), Scenario 3 (C), Scenario 4 (D) and Scenario 5 (E). Scenario 1: R_0_ = 1.65, hospitalization rate = 0.4%; no intervention; pre-existing immunity in place; Scenario 2 R_0_ = 1.80, hospitalization rate = 0.4%; no intervention; pre-existing immunity in place; Scenario 3: R_0_ = 1.65, hospitalization rate = 1.0%; no intervention; pre-existing immunity in place; Scenario 4: R_0_ = 1.65, hospitalization rate = 0.4%; 25% pre-vaccination; pre-existing immunity in place; Scenario 5: R_0_ = 1.65, hospitalization rate = 0.4%; no intervention; no pre-existing immunity.

**A)**

**
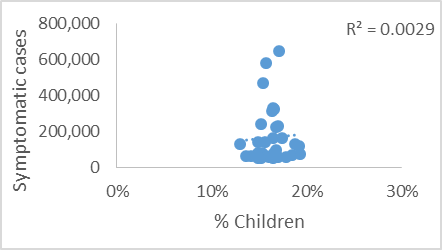
**

**B)**

**
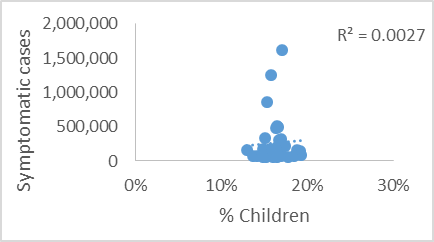
**

**C)**

**
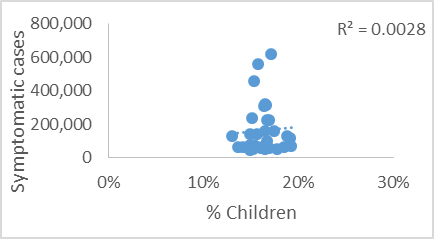
**

**D)**

**
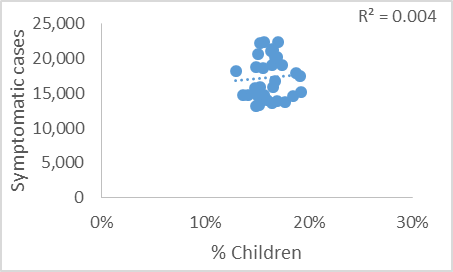
**

**E)**


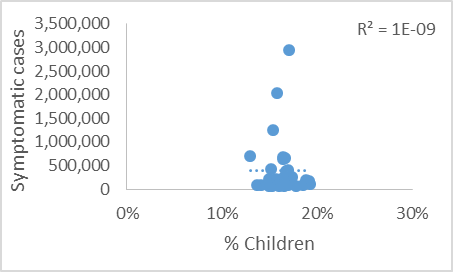


**Figure I.8.** Association between the percentage of the total population represented by children and overall number of acute hospital admissions. Negligible correlation in Scenario 1 (A), Scenario 2 (B), Scenario 3 (C), and Scenario 5 (E); mild correlation in Scenario 4 (D). Scenario 1: R_0_ = 1.65, hospitalization rate = 0.4%; no intervention; pre-existing immunity in place; Scenario 2 R_0_ = 1.80, hospitalization rate = 0.4%; no intervention; pre-existing immunity in place; Scenario 3: R_0_ = 1.65, hospitalization rate = 1.0%; no intervention; pre-existing immunity in place; Scenario 4: R_0_ = 1.65, hospitalization rate = 0.4%; 25% pre-vaccination; pre-existing immunity in place; Scenario 5: R_0_ = 1.65, hospitalization rate = 0.4%; no intervention; no pre-existing immunity.

**A)**

**
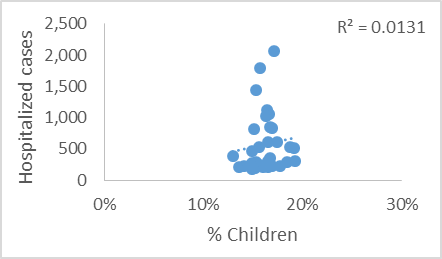
**

**B)**

**
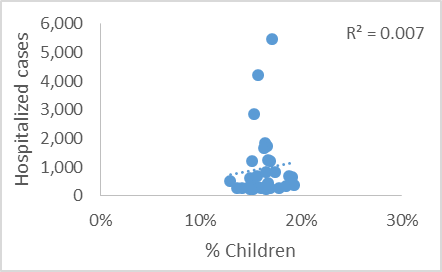
**

**C)**

**
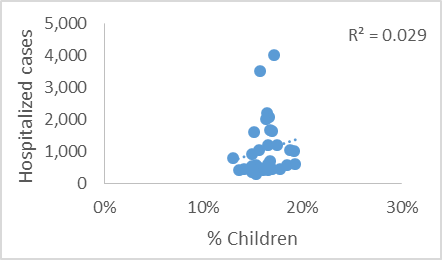
**

**D)**

**
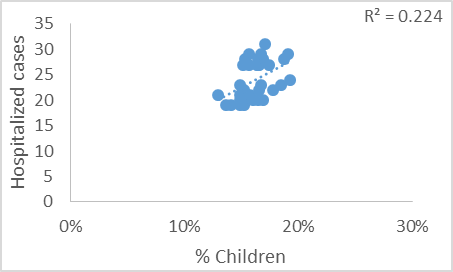
**

**E)**


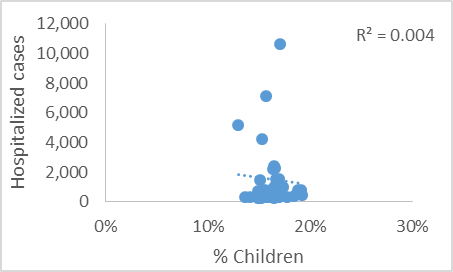


**Figure I.9.** Association between the percentage of the total population represented by children and overall number of ICU admissions. Negligible correlation in Scenario 1 (A), Scenario 2 (B), Scenario 3 (C), and Scenario 5 (E); moderate correlation in Scenario 4 (D). Scenario 1: R_0_ = 1.65, hospitalization rate = 0.4%; no intervention; pre-existing immunity in place; Scenario 2 R_0_ = 1.80, hospitalization rate = 0.4%; no intervention; pre-existing immunity in place; Scenario 3: R_0_ = 1.65, hospitalization rate = 1.0%; no intervention; pre-existing immunity in place; Scenario 4: R_0_ = 1.65, hospitalization rate = 0.4%; 25% pre-vaccination; pre-existing immunity in place; Scenario 5: R_0_ = 1.65, hospitalization rate = 0.4%; no intervention; no pre-existing immunity.

**A)**

**
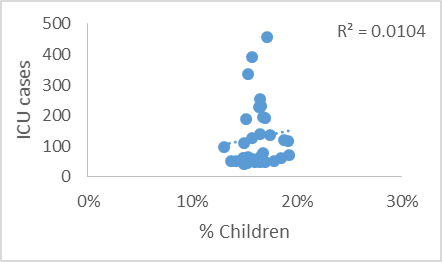
**

**B)**

**
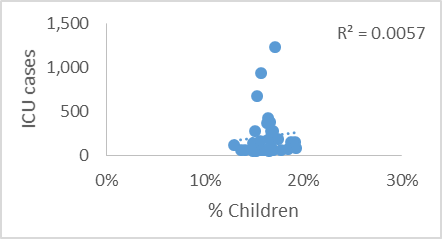
**

**C)**

**
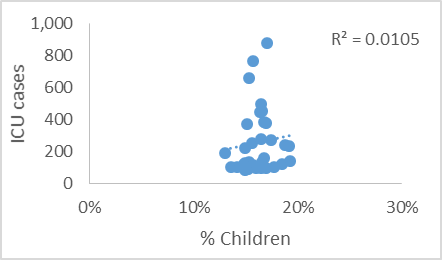
**

**D)**

**
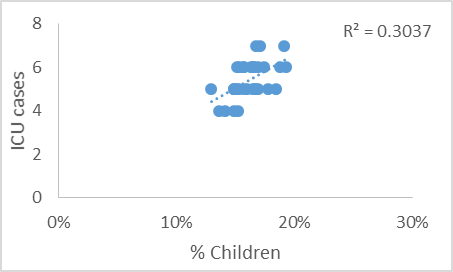
**

**E)**


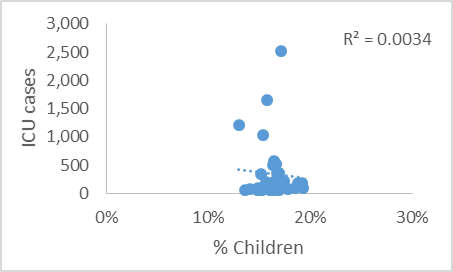


**Figure I.10.** Association between the percentage of the total population represented by children and peak acute-care demand (as percentage of total capacity). Weak correlation in Scenario 4 (D) and Scenario 5 (E); moderate correlation in Scenario 1 (A), Scenario 2 (B) and Scenario 3 (C). Scenario 1: R_0_ = 1.65, hospitalization rate = 0.4%; no intervention; pre-existing immunity in place; Scenario 2 R_0_ = 1.80, hospitalization rate = 0.4%; no intervention; pre-existing immunity in place; Scenario 3: R_0_ = 1.65, hospitalization rate = 1.0%; no intervention; pre-existing immunity in place; Scenario 4: R_0_ = 1.65, hospitalization rate = 0.4%; 25% pre-vaccination; pre-existing immunity in place; Scenario 5: R_0_ = 1.65, hospitalization rate = 0.4%; no intervention; no pre-existing immunity.

**A)**

**
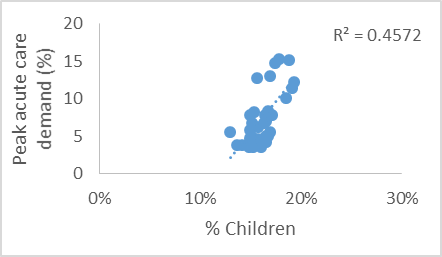
**

**B)**

**
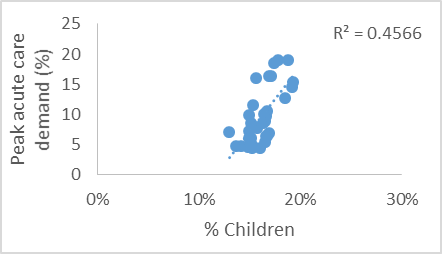
**

**C)**

**
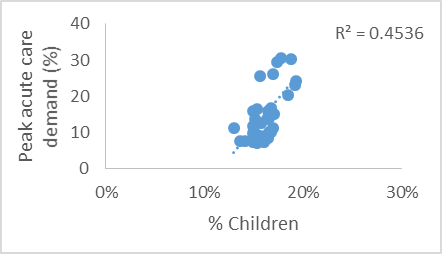
**

**D)**

**
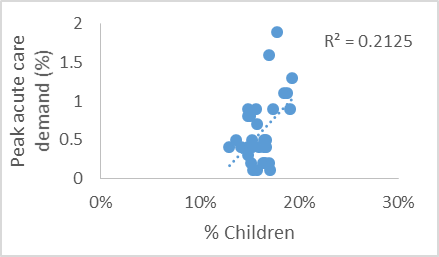
**

**E)**


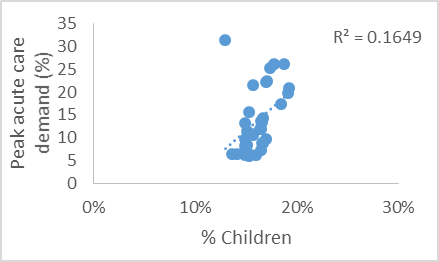


**Figure I.11.** Association between the percentage of the total population represented by children and peak ICU demand (as percentage of total capacity). Weak correlation in Scenario 1 (A), Scenario 2 (B), Scenario 3 (C) and Scenario 4 (D); moderate correlation in Scenario 5 (E). Scenario 1: R_0_ = 1.65, hospitalization rate = 0.4%; no intervention; pre-existing immunity in place; Scenario 2 R_0_ = 1.80, hospitalization rate = 0.4%; no intervention; pre-existing immunity in place; Scenario 3: R_0_ = 1.65, hospitalization rate = 1.0%; no intervention; pre-existing immunity in place; Scenario 4: R_0_ = 1.65, hospitalization rate = 0.4%; 25% pre-vaccination; pre-existing immunity in place; Scenario 5: R_0_ = 1.65, hospitalization rate = 0.4%; no intervention; no pre-existing immunity.

**A)**

**
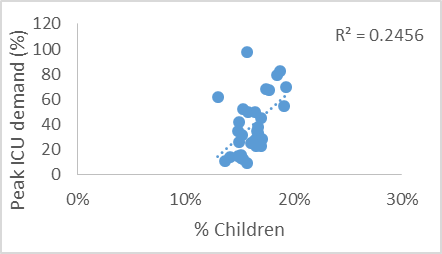
**

**B)**

**
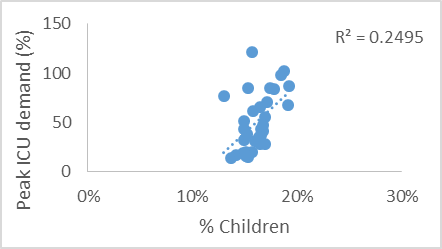
**

**C)**

**
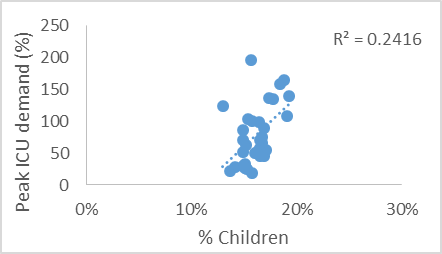
**

**D)**

**
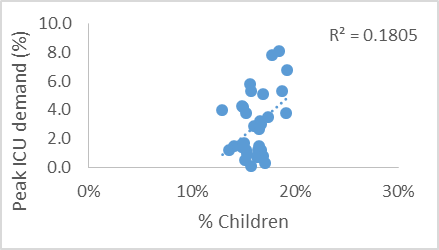
**

**E)**


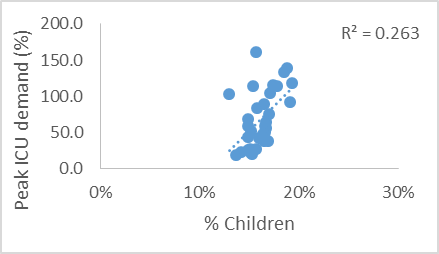


**Figure I.12.** Association between the percentage of the total population represented by children and overall mortality. Negligible correlation in Scenario 1 (A), Scenario 2 (B), Scenario 3 (C) and Scenario 5 (E); moderate correlation in Scenario 4 (E). Scenario 1: R_0_ = 1.65, hospitalization rate = 0.4%; no intervention; pre-existing immunity in place; Scenario 2 R_0_ = 1.80, hospitalization rate = 0.4%; no intervention; pre-existing immunity in place; Scenario 3: R_0_ = 1.65, hospitalization rate = 1.0%; no intervention; pre-existing immunity in place; Scenario 4: R_0_ = 1.65, hospitalization rate = 0.4%; 25% pre-vaccination; pre-existing immunity in place; Scenario 5: R_0_ = 1.65, hospitalization rate = 0.4%; no intervention; no pre-existing immunity.

**A)**

**
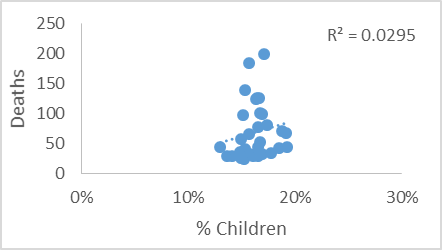
**

**B)**

**
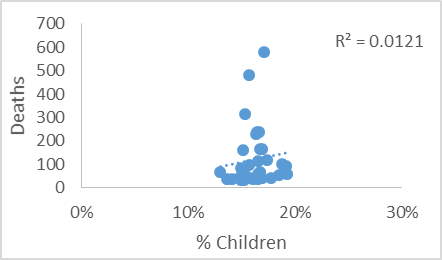
**

**C)**

**
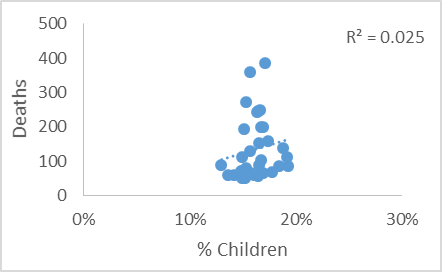
**

**D)**

**
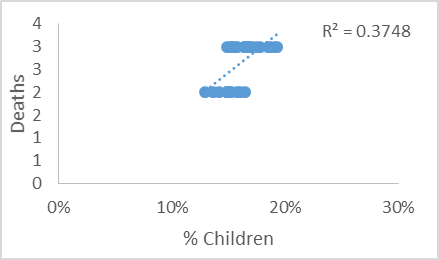
**

**E)**


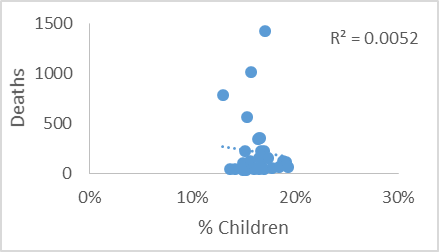


**Figure I.13.** Association between the percentage of the total population represented by young adults and overall number of symptomatic cases. Negligible correlation in Scenario 1 (A), Scenario 2 (B), Scenario 3 (C), Scenario 4 (D) and Scenario 5 (E). Scenario 1: R_0_ = 1.65, hospitalization rate = 0.4%; no intervention; pre-existing immunity in place; Scenario 2 R_0_ = 1.80, hospitalization rate = 0.4%; no intervention; pre-existing immunity in place; Scenario 3: R_0_ = 1.65, hospitalization rate = 1.0%; no intervention; pre-existing immunity in place; Scenario 4: R_0_ = 1.65, hospitalization rate = 0.4%; 25% pre-vaccination; pre-existing immunity in place; Scenario 5: R_0_ = 1.65, hospitalization rate = 0.4%; no intervention; no pre-existing immunity.

**A)**

**
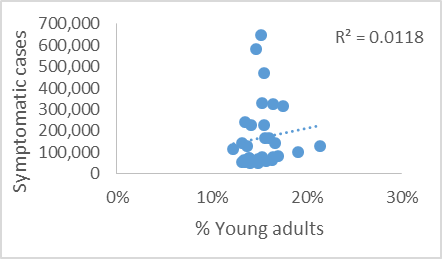
**

**B)**

**
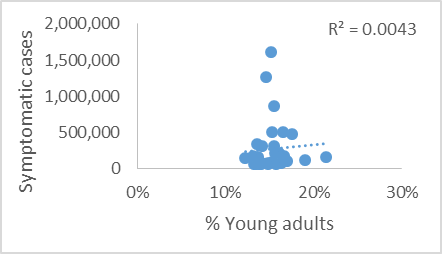
**

**C)**

**
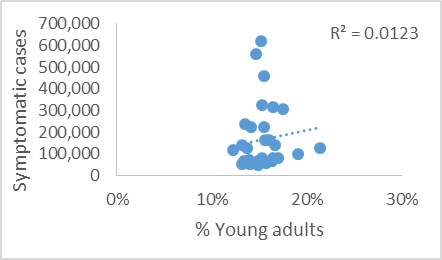
**

**D)**

**
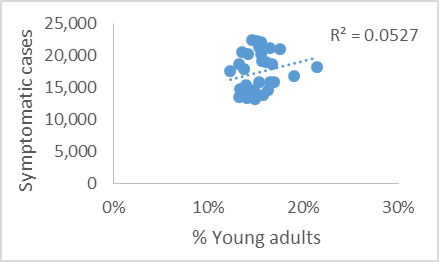
**

**E)**


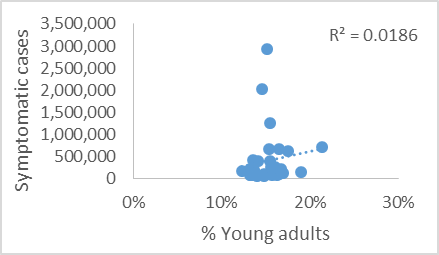


**Figure I.14.** Association between the percentage of the total population represented by young adults and overall number of acute hospital admissions. Negligible correlation in Scenario 1 (A), Scenario 2 (B), Scenario 3 (C), Scenario 4 (D) and Scenario 5 (E). Scenario 1: R_0_ = 1.65, hospitalization rate = 0.4%; no intervention; pre-existing immunity in place; Scenario 2 R_0_ = 1.80, hospitalization rate = 0.4%; no intervention; pre-existing immunity in place; Scenario 3: R_0_ = 1.65, hospitalization rate = 1.0%; no intervention; pre-existing immunity in place; Scenario 4: R_0_ = 1.65, hospitalization rate = 0.4%; 25% pre-vaccination; pre-existing immunity in place; Scenario 5: R_0_ = 1.65, hospitalization rate = 0.4%; no intervention; no pre-existing immunity.

**A)**

**
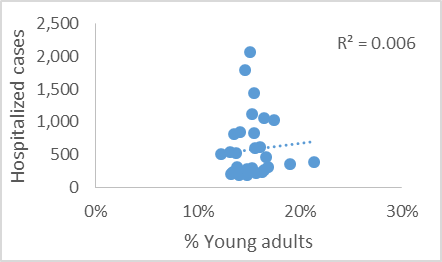
**

**B)**

**
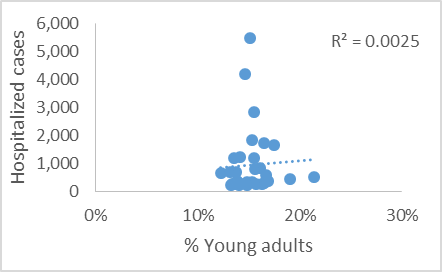
**

**C)
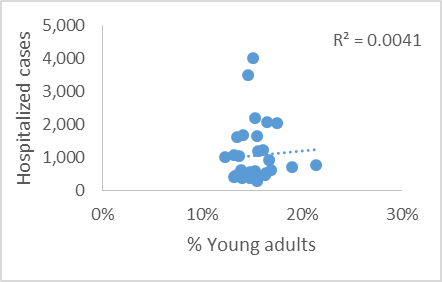
**

**D)**

**
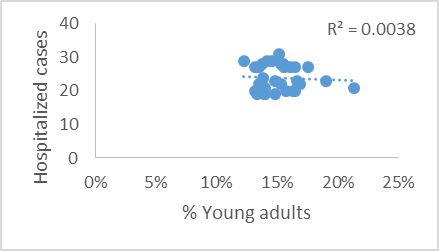
**

**E)**


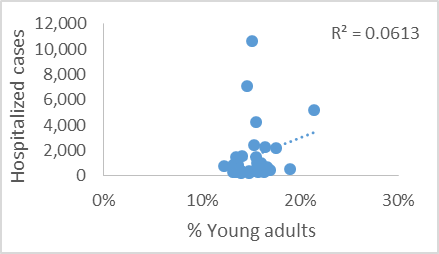


**Figure I.15.** Association between the percentage of the total population represented by young adults and overall number of ICU admissions. Negligible correlation in Scenario 1 (A), Scenario 2 (B), Scenario 3 (C), Scenario 4 (D) and Scenario 5 (E). Scenario 1: R_0_ = 1.65, hospitalization rate = 0.4%; no intervention; pre-existing immunity in place; Scenario 2 R_0_ = 1.80, hospitalization rate = 0.4%; no intervention; pre-existing immunity in place; Scenario 3: R_0_ = 1.65, hospitalization rate = 1.0%; no intervention; pre-existing immunity in place; Scenario 4: R_0_ = 1.65, hospitalization rate = 0.4%; 25% pre-vaccination; pre-existing immunity in place; Scenario 5: R_0_ = 1.65, hospitalization rate = 0.4%; no intervention; no pre-existing immunity.

**A)**

**
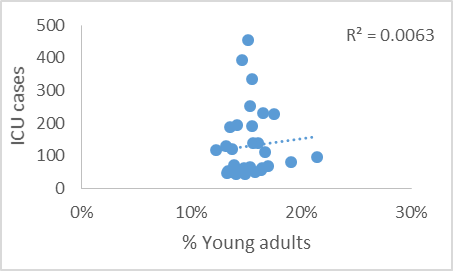
**

**B)**

**
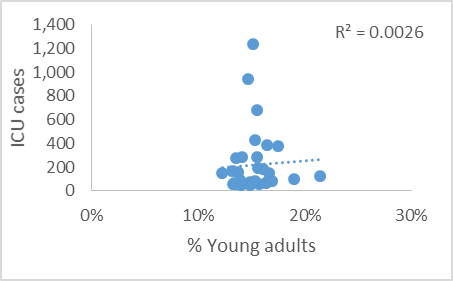
**

**C)**

**
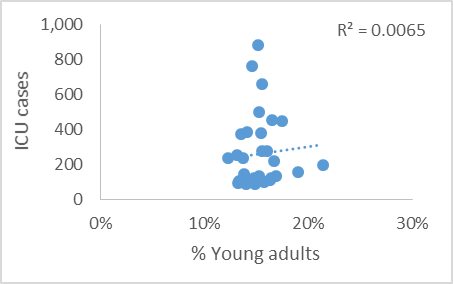
**

**D)**

**
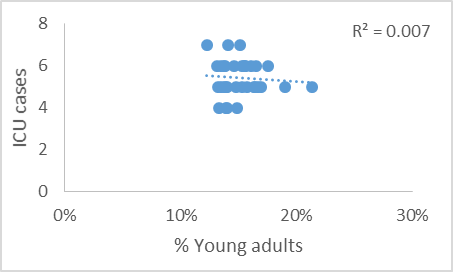
**

**E)**


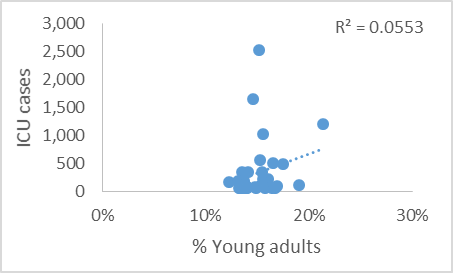


**Figure I.16.** Association between the percentage of the total population represented by young adults and peak acute-care demand (as a percentage of total capacity). Negligible correlation in Scenario 1 (A), Scenario 2 (B), Scenario 3 (C), Scenario 4 (D) and Scenario 5 (E). Scenario 1: R_0_ = 1.65, hospitalization rate = 0.4%; no intervention; pre-existing immunity in place; Scenario 2 R_0_ = 1.80, hospitalization rate = 0.4%; no intervention; pre-existing immunity in place; Scenario 3: R_0_ = 1.65, hospitalization rate = 1.0%; no intervention; pre-existing immunity in place; Scenario 4: R_0_ = 1.65, hospitalization rate = 0.4%; 25% pre-vaccination; pre-existing immunity in place; Scenario 5: R_0_ = 1.65, hospitalization rate = 0.4%; no intervention; no pre-existing immunity.

**A)**

**
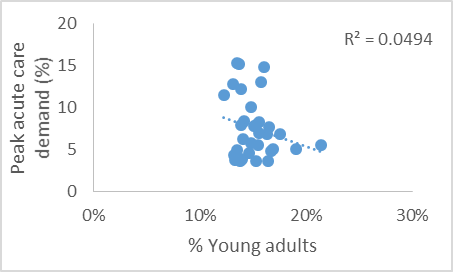
**

**B)**

**
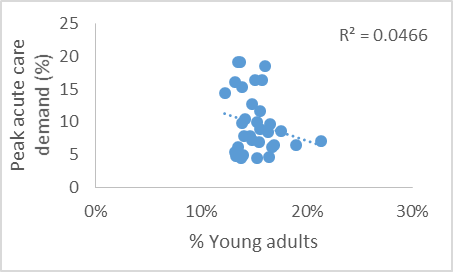
**

**C)**

**
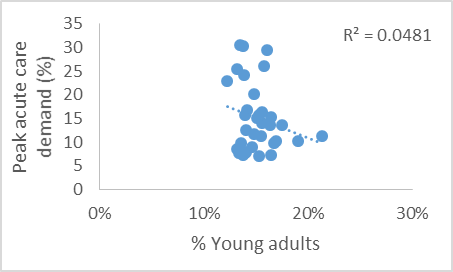
**

**D)**

**
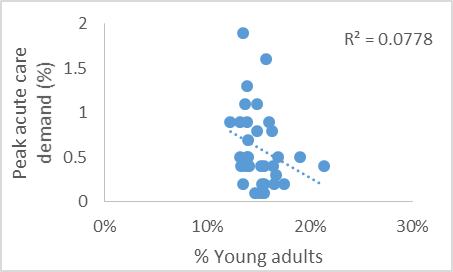
**

**E)**


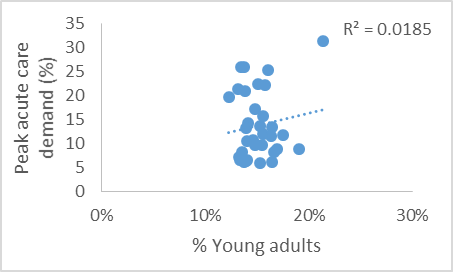


**Figure I.17.** Association between the percentage of the total population represented by young adults and peak ICU demand (as a percentage of total capacity). Negligible correlation in Scenario 1 (A), Scenario 2 (B), Scenario 3 (C), Scenario 4 (D) and Scenario 5 (E). Scenario 1: R_0_ = 1.65, hospitalization rate = 0.4%; no intervention; pre-existing immunity in place; Scenario 2 R_0_ = 1.80, hospitalization rate = 0.4%; no intervention; pre-existing immunity in place; Scenario 3: R_0_ = 1.65, hospitalization rate = 1.0%; no intervention; pre-existing immunity in place; Scenario 4: R_0_ = 1.65, hospitalization rate = 0.4%; 25% pre-vaccination; pre-existing immunity in place; Scenario 5: R_0_ = 1.65, hospitalization rate = 0.4%; no intervention; no pre-existing immunity.

**A)**

**
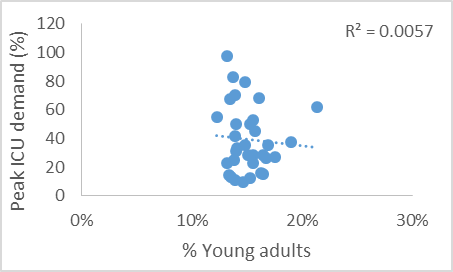
**

**B)**

**
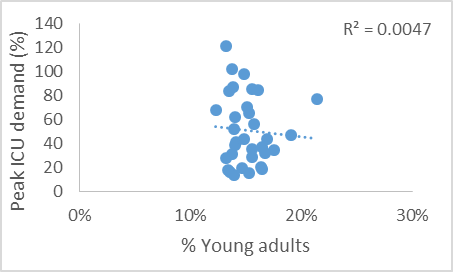
**

**C)**

**
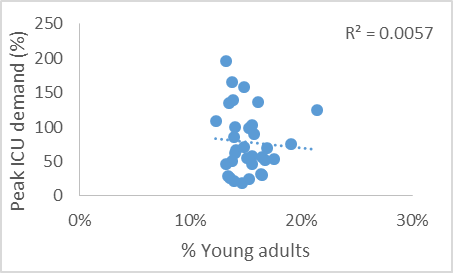
**

**D)**

**
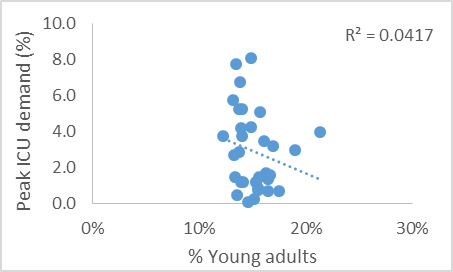
**

**E)**


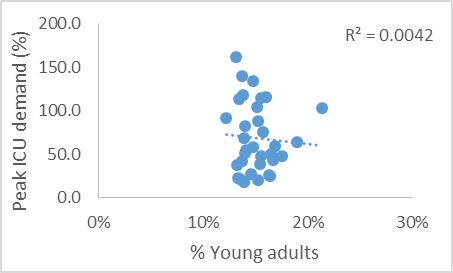


**Figure I.18.** Association between the percentage of the total population represented by young adults and total mortality. Negligible correlation in Scenario 1 (A), Scenario 2 (B), Scenario 3 (C), Scenario 4 (D) and Scenario 5 (E). Scenario 1: R_0_ = 1.65, hospitalization rate = 0.4%; no intervention; pre-existing immunity in place; Scenario 2 R_0_ = 1.80, hospitalization rate = 0.4%; no intervention; pre-existing immunity in place; Scenario 3: R_0_ = 1.65, hospitalization rate = 1.0%; no intervention; pre-existing immunity in place; Scenario 4: R_0_ = 1.65, hospitalization rate = 0.4%; 25% pre-vaccination; pre-existing immunity in place; Scenario 5: R_0_ = 1.65, hospitalization rate = 0.4%; no intervention; no pre-existing immunity.

**A)**

**
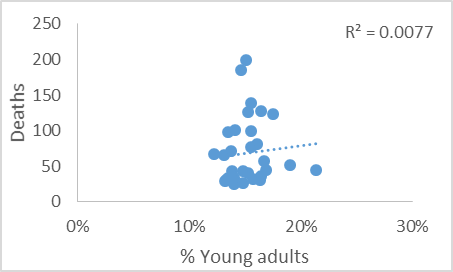
**

**B)**

**
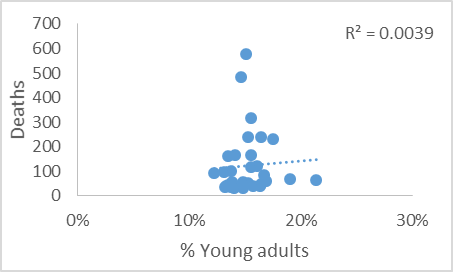
**

**C)**

**
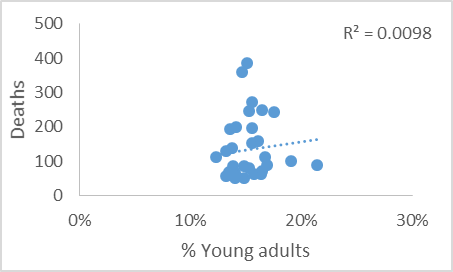
**

**D)**

**
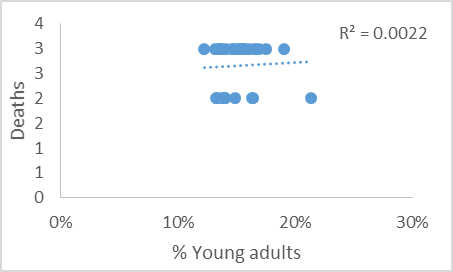
**

**E)**


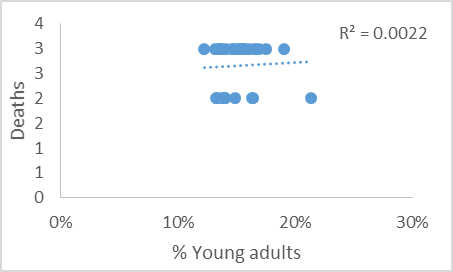


**Figure I.19.** Association between the percentage of the total population represented by adults and total symptomatic cases. Negligible correlation in Scenario 2 (B), Scenario 4 (D) and Scenario 5 (E); weak correlation in Scenario 1 (A) and Scenario 3 (C). Scenario 1: R_0_ = 1.65, hospitalization rate = 0.4%; no intervention; pre-existing immunity in place; Scenario 2 R_0_ = 1.80, hospitalization rate = 0.4%; no intervention; pre-existing immunity in place; Scenario 3: R_0_ = 1.65, hospitalization rate = 1.0%; no intervention; pre-existing immunity in place; Scenario 4: R_0_ = 1.65, hospitalization rate = 0.4%; 25% pre-vaccination; pre-existing immunity in place; Scenario 5: R_0_ = 1.65, hospitalization rate = 0.4%; no intervention; no pre-existing immunity.

**A)**

**
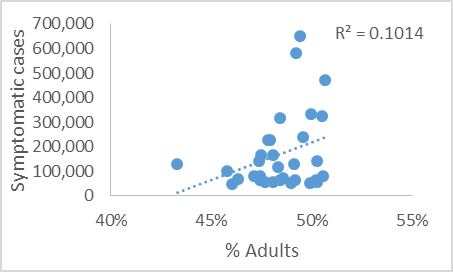
**

**B)**

**
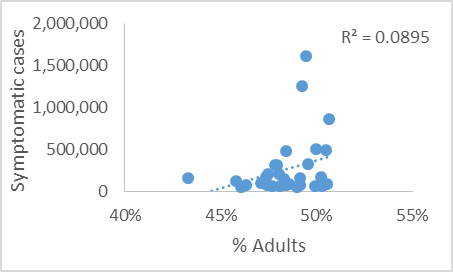
**

**C)**

**
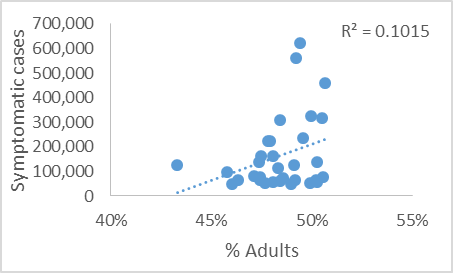
**

**D)**

**
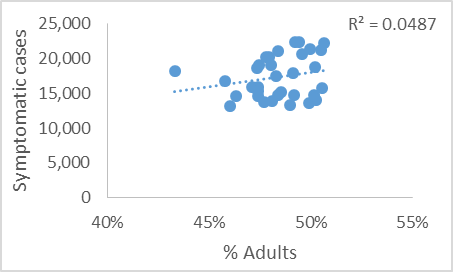
**

**E)**


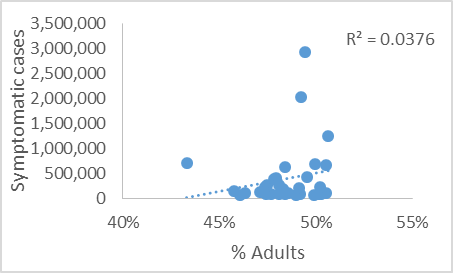


**Figure I.20.** Association between the percentage of the total population represented by adults and total acute hospital admissions. Negligible correlation in Scenario 2 (B), Scenario 3 (C), Scenario 4 (D) and Scenario 5 (E); weak correlation in Scenario 1 (A). Scenario 1: R_0_ = 1.65, hospitalization rate = 0.4%; no intervention; pre-existing immunity in place; Scenario 2 R_0_ = 1.80, hospitalization rate = 0.4%; no intervention; pre-existing immunity in place; Scenario 3: R_0_ = 1.65, hospitalization rate = 1.0%; no intervention; pre-existing immunity in place; Scenario 4: R_0_ = 1.65, hospitalization rate = 0.4%; 25% pre-vaccination; pre-existing immunity in place; Scenario 5: R_0_ = 1.65, hospitalization rate = 0.4%; no intervention; no pre-existing immunity.

**A)**

**
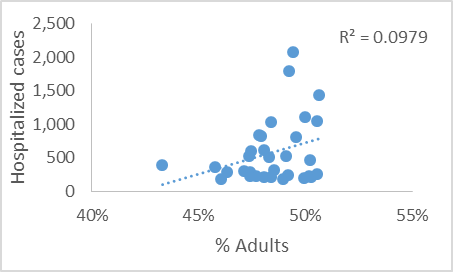
**

**B)**

**
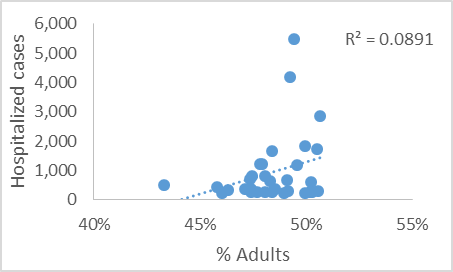
**

**C)**

**
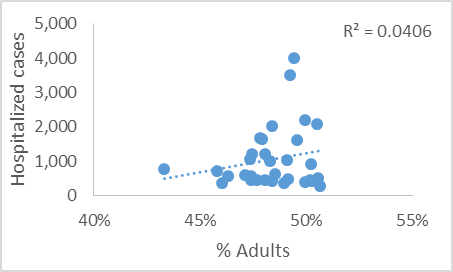
**

**D)**

**
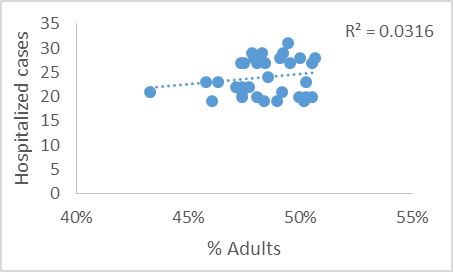
**

**E)**


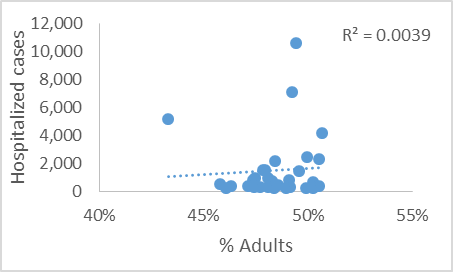


**Figure I.21.** Association between the percentage of the total population represented by adults and total ICU admissions. Negligible correlation in Scenario 4 (D) and Scenario 5 (E); weak correlation in Scenario 1 (A). Scenario 2 (B) and Scenario 3 (C). Scenario 1: R_0_ = 1.65, hospitalization rate = 0.4%; no intervention; pre-existing immunity in place; Scenario 2 R_0_ = 1.80, hospitalization rate = 0.4%; no intervention; pre-existing immunity in place; Scenario 3: R_0_ = 1.65, hospitalization rate = 1.0%; no intervention; pre-existing immunity in place; Scenario 4: R_0_ = 1.65, hospitalization rate = 0.4%; 25% pre-vaccination; pre-existing immunity in place; Scenario 5: R_0_ = 1.65, hospitalization rate = 0.4%; no intervention; no pre-existing immunity.

**A)**

**
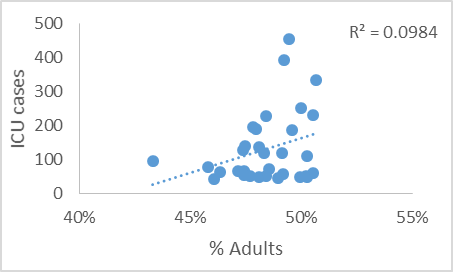
**

**B)**

**
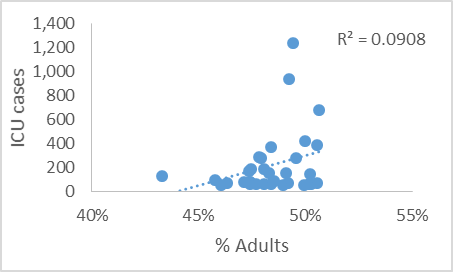
**

**C)**

**
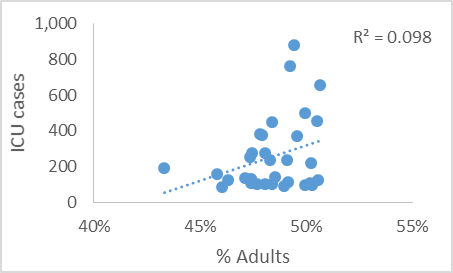
**

**D)**

**
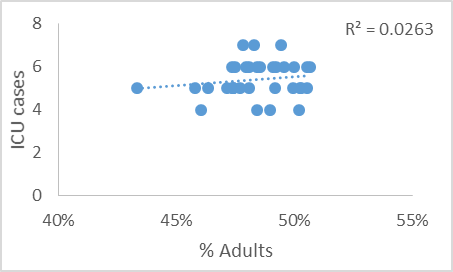
**

**E)**


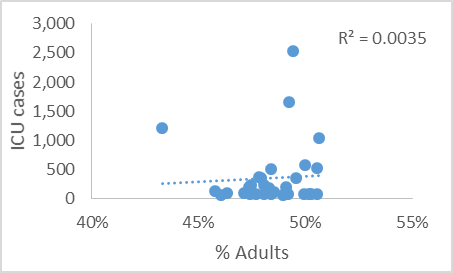


**Figure I.22.** Association between the percentage of the total population represented by adults and peak acute-care demand (as a percentage of total capacity). Negligible correlation in Scenario 1 (A), Scenario 2 (B) and Scenario 3 (C); weak correlation in Scenario 4 (D) and Scenario 5 (E). Scenario 1: R_0_ = 1.65, hospitalization rate = 0.4%; no intervention; pre-existing immunity in place; Scenario 2 R_0_ = 1.80, hospitalization rate = 0.4%; no intervention; pre-existing immunity in place; Scenario 3: R_0_ = 1.65, hospitalization rate = 1.0%; no intervention; pre-existing immunity in place; Scenario 4: R_0_ = 1.65, hospitalization rate = 0.4%; 25% pre-vaccination; pre-existing immunity in place; Scenario 5: R_0_ = 1.65, hospitalization rate = 0.4%; no intervention; no pre-existing immunity.

**A)**

**
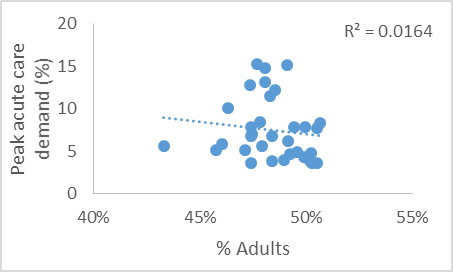
**

**B)**

**
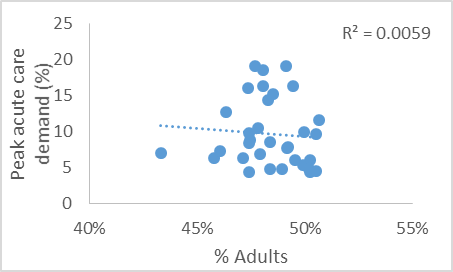
**

**C)**

**
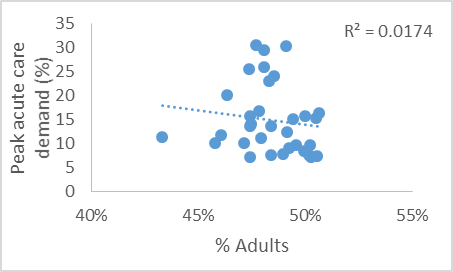
**

**D)**

**
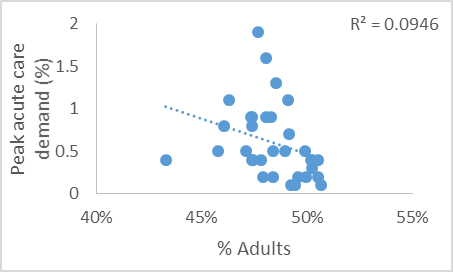
**

**E)**


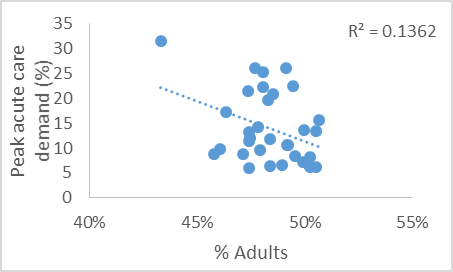


**Figure I.23.** Association between the percentage of the total population represented by adults and peak ICU demand (as a percentage of total capacity). Negligible correlation in Scenario 2 (B) and Scenario 5 (E); weak correlation in Scenario 1 (A), Scenario 3 (C) and Scenario 4 (D). Scenario 1: R_0_ = 1.65, hospitalization rate = 0.4%; no intervention; pre-existing immunity in place; Scenario 2 R_0_ = 1.80, hospitalization rate = 0.4%; no intervention; pre-existing immunity in place; Scenario 3: R_0_ = 1.65, hospitalization rate = 1.0%; no intervention; pre-existing immunity in place; Scenario 4: R_0_ = 1.65, hospitalization rate = 0.4%; 25% pre-vaccination; pre-existing immunity in place; Scenario 5: R_0_ = 1.65, hospitalization rate = 0.4%; no intervention; no pre-existing immunity.

**A)**

**
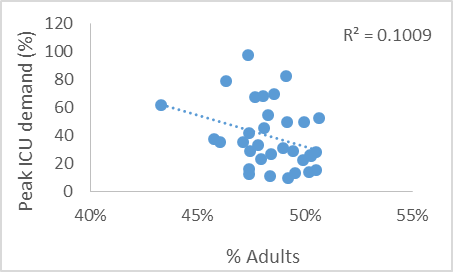
**

**B)**

**
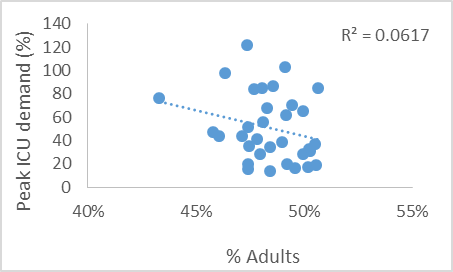
**

**C)**

**
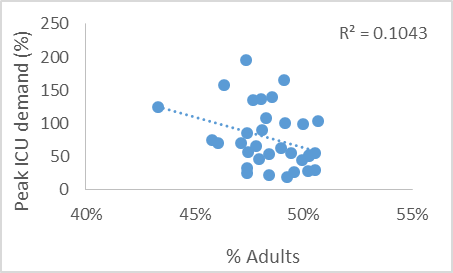
**

**D)**

**
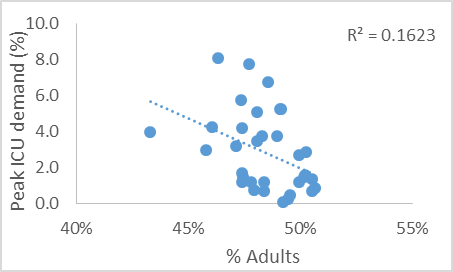
**

**E)**


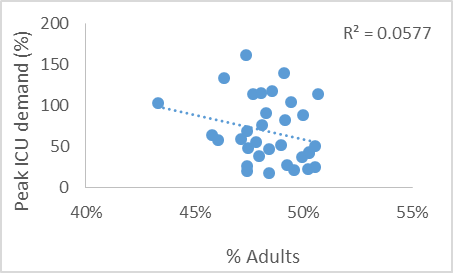


**Figure I.24.** Association between the percentage of the total population represented by adults and total mortality. Negligible correlation in Scenario 4 (D) and Scenario 5 (E); weak correlation in Scenario 1 (A), Scenario 2 (B) and Scenario 3 (C). Scenario 1: R_0_ = 1.65, hospitalization rate = 0.4%; no intervention; pre-existing immunity in place; Scenario 2 R_0_ = 1.80, hospitalization rate = 0.4%; no intervention; pre-existing immunity in place; Scenario 3: R_0_ = 1.65, hospitalization rate = 1.0%; no intervention; pre-existing immunity in place; Scenario 4: R_0_ = 1.65, hospitalization rate = 0.4%; 25% pre-vaccination; pre-existing immunity in place; Scenario 5: R_0_ = 1.65, hospitalization rate = 0.4%; no intervention; no pre-existing immunity.

**A)**

**
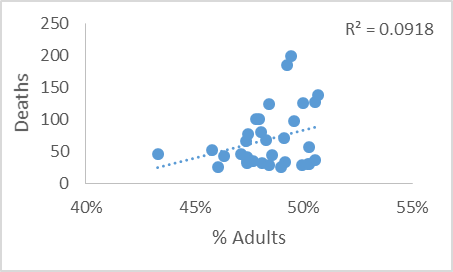
**

**B)**

**
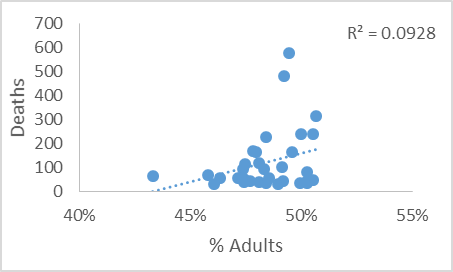
**

**C)**

**D)**

**E)**

**Figure I.25.** Association between the percentage of the total population represented by seniors and total symptomatic cases. Negligible correlation in Scenario 5 (E); weak correlation in Scenario 1 (A), Scenario 2 (B), Scenario 3 (C) and Scenario 4 (D). Scenario 1: R_0_ = 1.65, hospitalization rate = 0.4%; no intervention; pre-existing immunity in place; Scenario 2 R_0_ = 1.80, hospitalization rate = 0.4%; no intervention; pre-existing immunity in place; Scenario 3: R_0_ = 1.65, hospitalization rate = 1.0%; no intervention; pre-existing immunity in place; Scenario 4: R_0_ = 1.65, hospitalization rate = 0.4%; 25% pre-vaccination; pre-existing immunity in place; Scenario 5: R_0_ = 1.65, hospitalization rate = 0.4%; no intervention; no pre-existing immunity.

**A)**

**B)**

**C)**

**D)**

**E)**

**Figure I.26.** Association between the percentage of the total population represented by seniors and total acute hospital admissions. Negligible correlation in Scenario 5 (E); weak correlation in Scenario 1 (A), Scenario 2 (B), Scenario 3 (C) and Scenario 4 (D). Scenario 1: R_0_ = 1.65, hospitalization rate = 0.4%; no intervention; pre-existing immunity in place; Scenario 2 R_0_ = 1.80, hospitalization rate = 0.4%; no intervention; pre-existing immunity in place; Scenario 3: R_0_ = 1.65, hospitalization rate = 1.0%; no intervention; pre-existing immunity in place; Scenario 4: R_0_ = 1.65, hospitalization rate = 0.4%; 25% pre-vaccination; pre-existing immunity in place; Scenario 5: R_0_ = 1.65, hospitalization rate = 0.4%; no intervention; no pre-existing immunity.

**A)**

**B)**

**C)**

**D)**

**E)**

**Figure I.27.** Association between the percentage of the total population represented by seniors and total ICU admissions. Negligible correlation in Scenario 5 (E); weak correlation in Scenario 1 (A), Scenario 2 (B), Scenario 3 (C) and Scenario 4 (D). Scenario 1: R_0_ = 1.65, hospitalization rate = 0.4%; no intervention; pre-existing immunity in place; Scenario 2 R_0_ = 1.80, hospitalization rate = 0.4%; no intervention; pre-existing immunity in place; Scenario 3: R_0_ = 1.65, hospitalization rate = 1.0%; no intervention; pre-existing immunity in place; Scenario 4: R_0_ = 1.65, hospitalization rate = 0.4%; 25% pre-vaccination; pre-existing immunity in place; Scenario 5: R_0_ = 1.65, hospitalization rate = 0.4%; no intervention; no pre-existing immunity.

**A)**

**B)**

**C)**

**D)**

**E)**

**Figure I.28.** Association between the percentage of the total population represented by seniors and peak acute-care demand (as a percentage of total capacity). Negligible correlation in Scenario 1 (A), Scenario 2 (B), Scenario 3 (C), Scenario 4 (E) and Scenario 5 (E). Scenario 1: R_0_ = 1.65, hospitalization rate = 0.4%; no intervention; pre-existing immunity in place; Scenario 2 R_0_ = 1.80, hospitalization rate = 0.4%; no intervention; pre-existing immunity in place; Scenario 3: R_0_ = 1.65, hospitalization rate = 1.0%; no intervention; pre-existing immunity in place; Scenario 4: R_0_ = 1.65, hospitalization rate = 0.4%; 25% pre-vaccination; pre-existing immunity in place; Scenario 5: R_0_ = 1.65, hospitalization rate = 0.4%; no intervention; no pre-existing immunity.

**A)**

**B)**

**C)**

**D)**

**E)**

**Figure I.29.** Association between the percentage of the total population represented by seniors and peak ICU demand (as a percentage of total capacity). Negligible correlation in Scenario 1 (A), Scenario 2 (B), Scenario 3 (C), Scenario 4 (E) and Scenario 5 (E). Scenario 1: R_0_ = 1.65, hospitalization rate = 0.4%; no intervention; pre-existing immunity in place; Scenario 2 R_0_ = 1.80, hospitalization rate = 0.4%; no intervention; pre-existing immunity in place; Scenario 3: R_0_ = 1.65, hospitalization rate = 1.0%; no intervention; pre-existing immunity in place; Scenario 4: R_0_ = 1.65, hospitalization rate = 0.4%; 25% pre-vaccination; pre-existing immunity in place; Scenario 5: R_0_ = 1.65, hospitalization rate = 0.4%; no intervention; no pre-existing immunity.

**A)**

**B)**

**C)**

**D)**

**E)**

**Figure I.30.** Association between the percentage of the total population represented by seniors and total mortality. Negligible correlation in Scenario 5 (E); weak correlation in Scenario 1 (A), Scenario 2 (B), Scenario 3 (C) and Scenario 4 (D). Scenario 1: R_0_ = 1.65, hospitalization rate = 0.4%; no intervention; pre-existing immunity in place; Scenario 2 R_0_ = 1.80, hospitalization rate = 0.4%; no intervention; pre-existing immunity in place; Scenario 3: R_0_ = 1.65, hospitalization rate = 1.0%; no intervention; pre-existing immunity in place; Scenario 4: R_0_ = 1.65, hospitalization rate = 0.4%; 25% pre-vaccination; pre-existing immunity in place; Scenario 5: R_0_ = 1.65, hospitalization rate = 0.4%; no intervention; no pre-existing immunity.

**A)**

**B)**

**C)**

**D)**

**E)**

**Figure I.31.** Association between the number of acute-care hospital beds per 10,000 population and peak acute-care demand (as a percentage of total capacity). Weak correlation in Scenario 4 (D); strong correlation in Scenario 1 (A), Scenario 2 (B), Scenario 3 (C) and Scenario 5 (E). Scenario 1: R_0_ = 1.65, hospitalization rate = 0.4%; no intervention; pre-existing immunity in place; Scenario 2 R_0_ = 1.80, hospitalization rate = 0.4%; no intervention; pre-existing immunity in place; Scenario 3: R_0_ = 1.65, hospitalization rate = 1.0%; no intervention; pre-existing immunity in place; Scenario 4: R_0_ = 1.65, hospitalization rate = 0.4%; 25% pre-vaccination; pre-existing immunity in place; Scenario 5: R_0_ = 1.65, hospitalization rate = 0.4%; no intervention; no pre-existing immunity.

**A)**

**B)**

**C)**

**D)**

**E)**

**Figure I.32.** Association between the number of acute-care hospital beds per 10,000 population and peak ICU demand (as a percentage of total capacity). Weak correlation in Scenario 4 (D); strong correlation in Scenario 1 (A), Scenario 2 (B), Scenario 3 (C) and Scenario 5 (E). Scenario 1: R_0_ = 1.65, hospitalization rate = 0.4%; no intervention; pre-existing immunity in place; Scenario 2 R_0_ = 1.80, hospitalization rate = 0.4%; no intervention; pre-existing immunity in place; Scenario 3: R_0_ = 1.65, hospitalization rate = 1.0%; no intervention; pre-existing immunity in place; Scenario 4: R_0_ = 1.65, hospitalization rate = 0.4%; 25% pre-vaccination; pre-existing immunity in place; Scenario 5: R_0_ = 1.65, hospitalization rate = 0.4%; no intervention; no pre-existing immunity.

**A)**

**B)**

**C)**

**D)**

**E)**

**Figure I.33.** Association between the number of ICU beds per 10,000 population and peak acute-care demand (as a percentage of total capacity). Weak correlation in Scenario 4 (D); moderate correlation in Scenario 1 (A), Scenario 2 (B), Scenario 3 (C) and Scenario 5 (E). Scenario 1: R_0_ = 1.65, hospitalization rate = 0.4%; no intervention; pre-existing immunity in place; Scenario 2 R_0_ = 1.80, hospitalization rate = 0.4%; no intervention; pre-existing immunity in place; Scenario 3: R_0_ = 1.65, hospitalization rate = 1.0%; no intervention; pre-existing immunity in place; Scenario 4: R_0_ = 1.65, hospitalization rate = 0.4%; 25% pre-vaccination; pre-existing immunity in place; Scenario 5: R_0_ = 1.65, hospitalization rate = 0.4%; no intervention; no pre-existing immunity.

**A)**

**B)**

**C)**

**D)**

**E)**

**Figure I.34.** Association between the number of ICU beds per 10,000 population and peak ICU demand (as a percentage of total capacity). Moderate correlation in Scenario 4 (D); strong correlation in Scenario 1 (A), Scenario 2 (B), Scenario 3 (C) and Scenario 5 (E). Scenario 1: R_0_ = 1.65, hospitalization rate = 0.4%; no intervention; pre-existing immunity in place; Scenario 2 R_0_ = 1.80, hospitalization rate = 0.4%; no intervention; pre-existing immunity in place; Scenario 3: R_0_ = 1.65, hospitalization rate = 1.0%; no intervention; pre-existing immunity in place; Scenario 4: R_0_ = 1.65, hospitalization rate = 0.4%; 25% pre-vaccination; pre-existing immunity in place; Scenario 5: R_0_ = 1.65, hospitalization rate = 0.4%; no intervention; no pre-existing immunity.

**A)**

**B)**

**C)**

**D)**

**E)**
